# Mitochondrial complex IV defects induce metabolic and signaling perturbations that expose potential vulnerabilities in HCT116 cells

**DOI:** 10.1002/2211-5463.13398

**Published:** 2022-04-01

**Authors:** Oro Uchenunu, Alexander V. Zhdanov, Phillipe Hutton, Predrag Jovanovic, Ye Wang, Dmitry E. Andreev, Laura Hulea, David J. Papadopoli, Daina Avizonis, Pavel V. Baranov, Michael N. Pollak, Dmitri B. Papkovsky, Ivan Topisirovic

**Affiliations:** ^1^ Lady Davis Institute for Medical Research Jewish General Hospital Montréal Canada; ^2^ 5620 Department of Experimental Medicine McGill University Montreal Canada; ^3^ 8795 School of Biochemistry and Cell Biology University College Cork Ireland; ^4^ Shemyakin‐Ovchinnikov Institute of Bioorganic Chemistry Moscow Russia; ^5^ Belozersky Institute of Physico‐Chemical Biology Lomonosov Moscow State University Russia; ^6^ Département de Médecine Département de Biochimie et Médecine Moléculaire Université de Montréal Maisonneuve‐Rosemont Hospital Research Centre Canada; ^7^ 5620 Gerald Bronfman Department of Oncology McGill University Montreal Canada; ^8^ 5620 Goodman Cancer Research Centre McGill University Montreal Canada; ^9^ 5620 Department of Biochemistry McGill University Montreal Canada

**Keywords:** AKT, AMPK, cytochrome *C* oxidase, metabolism, mitochondrial dysfunction, mTOR, SCO2

## Abstract

Mutations in genes encoding cytochrome *c* oxidase (mitochondrial complex IV) subunits and assembly factors [e.g., synthesis of cytochrome *c* oxidase 2 (SCO2)] are linked to severe metabolic syndromes. Notwithstanding that *SCO2* is under transcriptional control of tumor suppressor p53, the role of mitochondrial complex IV dysfunction in cancer metabolism remains obscure. Herein, we demonstrate that the loss of *SCO2* in HCT116 colorectal cancer cells leads to significant metabolic and signaling perturbations. Specifically, abrogation of *SCO2* increased NAD^+^ regenerating reactions and decreased glucose oxidation through citric acid cycle while enhancing pyruvate carboxylation. This was accompanied by a reduction in amino acid levels and the accumulation of lipid droplets. In addition, *SCO2* loss resulted in hyperactivation of the insulin‐like growth factor 1 receptor (IGF1R)/AKT axis with paradoxical downregulation of mTOR signaling, which was accompanied by increased AMP‐activated kinase activity. Accordingly, abrogation of *SCO2* expression appears to increase the sensitivity of cells to IGF1R and AKT, but not mTOR inhibitors. Finally, the loss of *SCO2* was associated with reduced proliferation and enhanced migration of HCT116 cells. Collectively, herein we describe potential adaptive signaling and metabolic perturbations triggered by mitochondrial complex IV dysfunction.

## Graphical abstract

In this study, we provide evidence suggesting potential adaptive mechanisms that are activated in response to dysfunction of mitochondrial complex IV in HCT116 colon cancer cell line. These mechanisms involve dramatic metabolic reprogramming to compensate for the disruption of mitochondrial functions that is accompanied by the activation of the IGF1R/AKT axis, increased AMPK activity, and paradoxical downregulation of mTORC1 signaling.

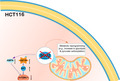

**Abbreviations**
4E‐BP1: eukaryotic translation initiation factor 4E‐binding protein 1
ACC: acetyl‐CoA carboxylase
AMPK: AMP‐activated kinase
CAC: citric acid cycle
COA6: cytochrome *c* oxidase assembly factor 6
COX: cytochrome *c* oxidase
DHAP: dihydroxyacetone phosphate
eIF2: eukaryotic translation initiation factor 2
EMT: epithelial–mesenchymal transition
FA: fatty acid
HIF: hypoxia‐inducible factor
HK2: hexokinase 2
IDH: isocitrate dehydrogenase
IGF1R: insulin‐like growth factor 1 receptor
MEF: mouse embryonic fibroblast
MTOR: mechanistic target of rapamycin
OXPHOS: oxidative phosphorylation
PDH: pyruvate dehydrogenase
PDK: pyruvate dehydrogenase kinase
ROS: reactive oxygen species
rpS6: ribosomal protein S6
SCO2: synthesis of cytochrome *c* oxidase 2
WT: wildtype

Driven by genetic, epigenetic, environmental, and other factors, malfunctions of citric acid cycle (CAC) enzymes, oxidative phosphorylation (OXPHOS) supercomplexes, and other mitochondrial proteins have been implicated in neoplastic transformation, tumor progression, and/or therapeutic responses [1, 2]. This is exemplified by inactivation of fumarate hydratase (FH), succinate dehydrogenase (SDH), or neomorphic mutations in isocitrate dehydrogenase (IDH) that result in accumulation of fumarate, succinate, and 2‐hydroxyglutarate, respectively [3, 4, 5, 6, 7]. These metabolites are thought to contribute to tumorigenesis and tumor progression by interfering with several classes of α‐ketoglutarate‐dependent enzymes that govern key cellular functions [8]. Cytochrome *c* oxidase (COX) assembly protein 2 (SCO2) is essential to the functioning of COX (mitochondrial complex IV). The loss of function or deletion of *SCO2* leads to abrogation of COX assembly and activity [9].

Though non‐lethal diseases associated with deficiencies of COX are rare [10, 11], cell models bearing the relevant mutations are useful for understanding metabolic re‐arrangements caused by mitochondrial complex IV deficiencies. To this end, studies in colon cancer HCT116 cell line devoid of *SCO2* (HCT116 *SCO2* ^−/−^ cells) showed that COX deficiency is associated with a decrease in O_2_ consumption and slower proliferation[12, 13, 14]. In addition, SCO2‐deficient cells exhibit an increase in NADH, reactive oxygen species (ROS), and glycolysis, which are accompanied by reversed activity of F_1_Fo ATP synthase [12, 13, 14]. These perturbations are paralleled by dramatic changes in gene expression including induction of the factors implicated in epithelial‐to‐mesenchymal transition (EMT) [12, 13, 14, 15]. Importantly, HCT116 *SCO2* ^−/−^ cells are more resilient to hypoxia than their SCO2‐proficient counterparts [13], which in conjunction with induction of EMT phenotypes, suggests that dysfunction of mitochondrial complex IV may play a central role in metastatic progression. *SCO2* expression is regulated by p53 [12]. Metabolic perturbations caused by the loss of *TP53* function are thus thought to be at least in part mediated by the downregulation of *SCO2* [12]. The loss of p53 function was reported to result in the reduction of OXPHOS and a compensatory increase in glycolysis [12], which is thought to be mediated by an increase in hexokinase 2 (HK2) [16] and phosphoglycerate mutase [17] and the reduction in TIGAR levels [18]. Deletion of the *SCO2* gene in HCT116 recapitulates the metabolic rewiring toward glycolysis that is observed in p53‐deficient cells [12]. However, it was also reported that wildtype (WT) *TP53* glioma and colon cancer cells adapt to hypoxia by at least in part modulating SCO2 expression [19]. Moreover, the correlation between SCO2 levels and cancer prognosis remains unclear. For instance, low expression of *SCO2* was associated with poor prognosis in breast and ovarian cancer patients [20, 21, 22]. In contrast, TCGA analysis revealed amplification and subsequent increase in SCO2 levels in metastatic cancers harboring *TP53* mutations, wherein high SCO2 levels correlated with poor prognosis [23]. Collectively, these findings demonstrate that notwithstanding that SCO2 is likely to play a major role in p53‐dependent metabolic reprogramming and thus determine the fate of cancer cells, the underpinning mechanisms and associated clinical correlates remain incompletely understood. In the attempt to address these gaps in knowledge, we systematically examined metabolic perturbations induced by *SCO2* loss in HCT116 cells. As expected, dysfunction of mitochondrial complex IV in *SCO2* ^−/−^ HCT116 cells resulted in dramatic metabolic reprogramming including elevated dependence on glucose and altered pyruvate and amino acid metabolism. These metabolic perturbations were accompanied by increased migration and sustained proliferation under extremely low (< 0.1%) oxygen levels. Accordingly, *SCO2* deletion in HCT116 cells increases survival under hypoxia compared with the WT cells [13]. Moreover, the loss of *SCO2* resulted in activation of insulin‐like growth factor 1 receptor (IGF1R)/AKT axis and elevated sensitivity to the IGF1R and AKT inhibitors.

## Results

### *SCO2* loss results in metabolic rewiring of HCT116 cells that is characterized by increased NAD^+^ regeneration

**Fig. 1:**
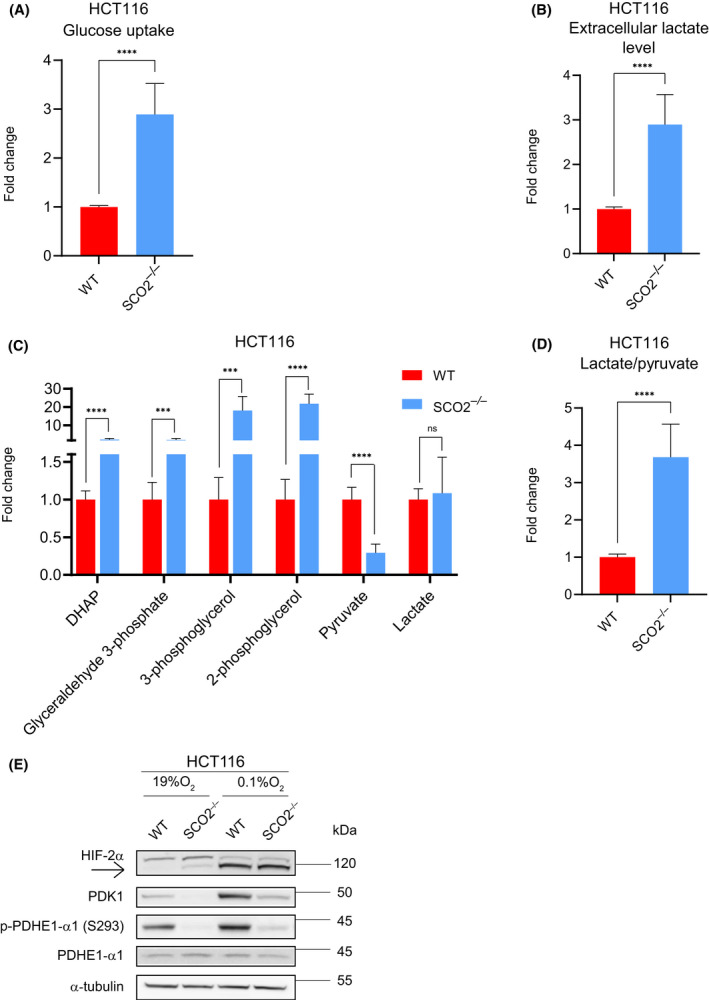
*SCO2* loss in HCT116 cells rewires glucose metabolism while decreasing PDHE1‐α1 phosphorylation. (A, B) Glucose uptake (A) and extracellular lactate levels (B) were determined using BioProfiler analyzer. Data are represented as mean fold change relative to the control, WT HCT116 cells (set to 1) ± standard deviation (SD). ****P < 0.0001 (Unpaired two‐tailed *t*‐test; *n* = 5 independent experiments with three technical replicates in each). (C) Intracellular levels of indicated metabolites in WT or *SCO2* ^−/−^HCT116 cells. Metabolite levels were monitored by GC‐MS. Data are presented as mean fold change relative to WT HCT116 cells (set to 1). Bars represent standard deviation values. n.s.‐non‐significant, ***P < 0.001, ****P < 0.0001 (Unpaired two‐tailed *t*‐test; *n* = 3 independent experiments with three technical replicates in each). (D) Intracellular lactate/pyruvate ratio in WT or HCT116 *SCO2* ^−/−^ cells. Intracellular lactate and pyruvate levels were determined by GC‐MS. Obtained lactate/pyruvate ratio in WT HCT116 cells was set to 1. Data are presented as mean fold change of *n* = 3 independent experiments with three technical replicates in each. Bars represent standard deviation values. ****P < 0.0001 (Unpaired two‐tailed *t*‐test). (E) Levels and phosphorylation status of indicated proteins in WT or *SCO2* ^−/−^ HCT116 cells were monitored by Western blotting. α‐tubulin served as a loading control. As indicated, cells were grown for 10 days under 19% and 0.1% O_2_. Shown are representative Western blots from three independent experiments. Quantifications of Western Blots are shown in Fig. S9.

Mitochondrial complex IV deficiency caused by *SCO2* loss leads to impairment of the electron transport chain, decreased pyruvate oxidation, and increased lactate production [13, 14]. To identify metabolic pathways that are altered by abrogation of *SCO2*, we first quantified intracellular and extracellular steady‐state levels of key metabolites. As expected, the loss or depletion of *SCO2* in HCT116 or A549 cells, respectively, increased glucose uptake (Fig. 1A, Fig. S1A), extracellular lactate levels (Fig. 1B, Fig. S1B), and glycolytic intermediates including dihydroxyacetone phosphate (DHAP), 3‐phosphoglycerol (3‐PG), and 2‐phosphoglycerol (2‐PG) (Fig. 1C). This is in agreement with previous observations that *SCO2* deletion or OXPHOS inhibition using biguanides increases levels of 3‐PG and lactate production in order to regenerate NAD^+^ necessary for maintaining glycolysis [13, 24, 25]. The re‐introduction of exogenous *SCO2* into *SCO2* ^−/−^ HCT116 cells attenuated increased glucose uptake and lactate secretion thus confirming that SCO2 suppresses glycolysis (Fig. S1C,D). Of note, notwithstanding repeated attempts, we could not achieve the rescue of *SCO2* expression to the levels of the endogenous protein (Fig. S1E). Taken together, the loss of *SCO2* in HCT116 cells increases their dependence on glucose metabolism.

**Fig. 2:**
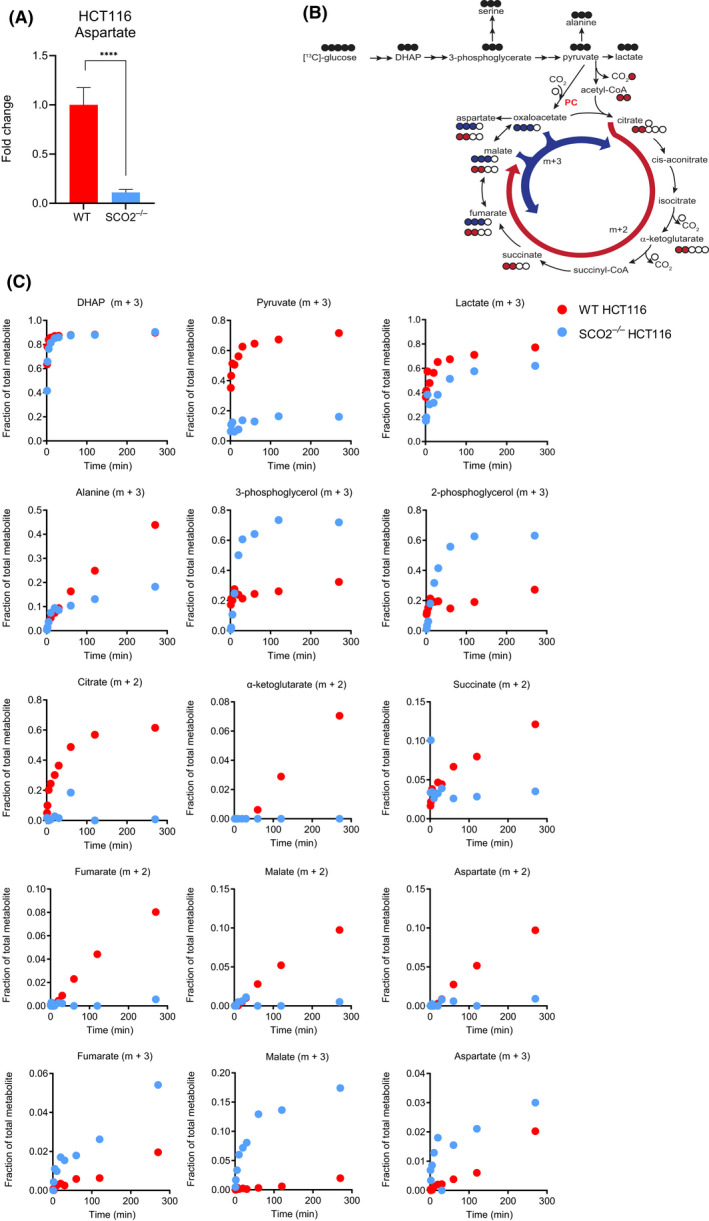
*SCO2* loss in HCT116 cells perturbs glycolysis, CAC, and aspartate synthesis. (A) Intracellular levels of aspartate in WT and *SCO2* ^−/−^ HCT116 cells. Aspartate levels were determined by GC‐MS. Results are represented as mean fold change ± SD in which the values calculated for WT HCT116 cells were set to 1. ****P < 0.0001 (Unpaired two‐tailed *t*‐test; *n* = 3 independent experiments with three technical replicates in each). (B) Schematic diagram of ^13^C incorporation into metabolites after incubation with ^13^C_6_‐glucose. Red arrow represents the incorporation of two ^13^C units via the oxidative carboxylation of CAC. Blue arrow represents the carboxylation of pyruvate via pyruvate carboxylase (PC) whereby resulting metabolites have three ^13^C units. (C) Mass spectrometry analysis of glucose‐derived metabolites (glucose flux) in *SCO2* ^−/−^ and WT HCT116 cells. Cells were grown in the presence of ^13^C_6_‐glucose for 1, 2, 5, 10, 20, 30, 60, 120 and 270 min. Representative plots of three independent experiments are shown.

In contrast to DHAP, pyruvate levels were found to be lower in *SCO2* ^−/−^ relative to WT HCT116 cells (Fig. 1C). This is likely due to the diversion of carbon units from the glycolytic pathway to glycerol biosynthesis via 3‐phosphoglycerol (3‐PG) as 3‐PG was found to be higher in *SCO2* ^−/−^ than in WT HCT116 cells (Fig. 1C). This is indicative of enhanced cytosolic glycerol‐3‐phosphate dehydrogenase (GPD1) activity which not only produces glycerol but also NAD^+^ [26]. We hypothesize that this reaction, in addition to the conversion of pyruvate to lactate, is necessary for maintaining glycolysis in the HCT116 *SCO2* ^−/−^ cells. Indeed, the intracellular lactate/pyruvate ratio, which is traditionally used as a proxy for cytosolic NADH/NAD^+^ ratio and glycolytic activity [27], was elevated in the *SCO2* ^−/−^ as compared to WT HCT116 cells (Fig. 1D). Since pyruvate oxidation is initiated by the pyruvate dehydrogenase (PDH), we investigated whether the *SCO2* status affects the levels and/or phosphorylation of PDH subunits. PDH kinases (PDK1–4) when stimulated by ATP, acetyl‐CoA or NADH phosphorylate and inactivate PDH thus deflecting pyruvate from CAC under conditions of mitochondrial dysfunction and hypoxia [28]. Surprisingly, we noted that both PDK1 levels and the phosphorylation of PDH subunit PDHE1‐α1 (S293) are decreased in *SCO2* ^−/−^ cells relative to WT HCT116 cells in both normoxia and hypoxia (Fig. 1E). This was despite the fact that NADH levels are higher in *SCO2* ^−/−^ than in WT HCT116 cells [13]. Considering that PDK1 expression is stimulated by hypoxia‐inducible factor (HIF)‐1 and 2α, we next determined HIF‐2α levels. HIF‐2α induction under severe hypoxia (<0.1% O_2_) was comparable between the cell lines (Fig. 1E). PDK1 levels and PDHE1‐α1 phosphorylation were, however, more strongly induced in WT as compared to HCT116 *SCO2* ^−/−^ cells (Fig. 1E). Collectively, these findings show that although the loss of *SCO2* increased NADH levels [13], PDK1 activity is reduced in *SCO2* ^−/−^ HCT116 cells as compared to WT HCT116 cells. Hence, although pyruvate oxidation may be altered in *SCO2* ^−/−^ HCT116 cells, this cannot be explained by NADH‐mediated regulation of PDK1 activity. Recent studies demonstrated that one of the major functions of mitochondrial respiration is to produce a sufficient amount of aspartate required to drive the proliferation of cancer cells [29, 30]. To this end, it is thought that most of cellular aspartate is derived from oxaloacetate produced in oxidative CAC [31]. Consistent with the disruption of the oxidative CAC in *SCO2* ^−/−^ HCT116 cells, steady‐state aspartate levels were dramatically reduced in *SCO2* ^−/−^ as compared to WT HCT116 cells (Fig. 2A). However, stable isotope tracer analysis (SITA) using ^13^C‐labeled glucose revealed that in *SCO2* ^−/−^ HCT116 cells, pyruvate may primarily undergo carboxylation followed by transamination to produce aspartate as illustrated by an increase in m + 3 fractions of malate, fumarate, and aspartate isotopomers (Fig. 2B,C). In turn, the levels of the m + 2 citrate, α‐KG, fumarate, and malate isotopomers were markedly reduced in *SCO2* ^−/−^ compared with WT HCT116 cells (Fig. 2C). These findings suggest that increased carboxylation of pyruvate may represent a compensatory mechanism for a decrease in CAC‐derived aspartate in *SCO2* ^−/−^ HCT116 cells. This, in addition to increased lactate production and contribution of 3‐PG to glycerol synthesis, may explain the reduction in pyruvate levels in SCO2‐deficient relative to WT HCT116 cells.

### Abrogation of *SCO2* in HCT116 cells leads to alterations in acetyl‐CoA and lipid metabolism

**Fig. 3:**
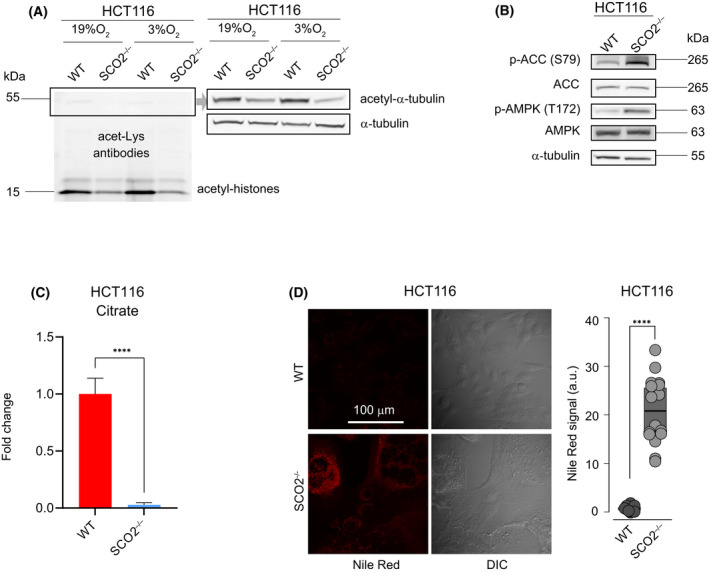
Complex IV deficiency alters FA metabolism in HCT116 cells. (A) Western blot analysis of α–tubulin (Lys 40) and total histone acetylation levels in WT and *SCO2* ^−/−^ HCT116 cells. Representative blots of two independent replicates are shown (B). Phosphorylation status and levels of indicated proteins were assessed in WT and *SCO2* ^−/−^ HCT116 by Western blotting. α‐tubulin served as a loading control. Shown are representative blots of three independent experiments. (C) Intracellular levels of citrate in WT and *SCO2* ^−/−^ HCT116 cells. Citrate levels were determined by GC‐MS. Results are represented as mean fold change ± SD whereby values obtained for WT HCT116 cells were set to 1. ****P < 0.0001 (Unpaired two‐tailed *t*‐test; *n* = 3 independent experiments with three technical replicates in each). (D) Confocal microscopy of lipid droplets in WT and *SCO2* ^−/−^ HCT116 cells using Nile Red staining. Fluorescence images are stacks of 5 focal planes taken with a 0.5 µm step, with single‐plane DIC images on the right (*n* = 3 independent experiments). Results of a representative experiment (*N* = 16 cells for each cell line) are normalized to the mean fluorescence intensity in WT HCT116 cells and shown as individual data points in arbitrary units (a.u.); mean value in WT HCT116 cells was set to 1 a.u. ****P < 0.0001 (Unpaired 2‐tailed *t*‐test). Quantifications of Western Blots are shown in Fig. S9.

Pyruvate is oxidized through PDH to produce acetyl‐coenzyme A (acetyl‐CoA), a 2‐carbon unit that enters the CAC and yields NADH [32]. Notwithstanding observed reduction in PDK1 activity in HCT116 cells devoid of SCO2 (Fig. 1E), PDH is sensitive to product inhibition by NADH [33]. Considering that NADH levels are elevated in *SCO2* ^−/−^ vs WT HCT116 cells [13] while SCO2 loss decreased pyruvate oxidation through the CAC (Fig. 2B,C), we hypothesized that SCO2 deletion is paralleled by a decrease in acetyl‐CoA levels. Despite our inability to measure acetyl‐CoA directly, we analyzed the effects of *SCO2* loss on acetyl‐CoA‐dependent pathways. Herein, we observed decreased levels of histone and α‐tubulin lysine *N*‐acetylation in *SCO2* ^−/−^ relative to WT HCT116 cells in both normoxia and moderate hypoxia (3% O_2_) (Fig. 3A). Thus, although the phosphorylation of S293 residue of PDHE1‐α1 subunit often negatively correlates with PDH activity, this is not the case for HCT116 *SCO2* ^−/−^ cells. Moreover, PDH can be inhibited when phosphorylated at either S232 or S300 residues of PDHE1‐α1 subunit by PDK1–4 [34, 35, 36]. Considering that acetyl‐CoA carboxylation which generates malonyl‐CoA is the rate‐limiting step in fatty acid (FA) synthesis [37], we next monitored acetyl‐CoA carboxylase (ACC) activity. Relative to WT, *SCO2* ^−/−^ HCT116 cells exhibited increased activity of AMP‐activated protein kinase (AMPK) that phosphorylates (S79) and inactivates ACC (Fig. 3B). In addition, the levels of citrate, which allosterically activates ACC, were markedly decreased in *SCO2* ^−/−^ as compared to WT HCT116 cells (Fig. 3C). Reduction in FA synthesis in *SCO2* ^−/−^ HCT116 as compared to WT cells also aligns with the increase in both the steady‐state and m + 3 levels of 2‐ and 3‐PG, which are triacylglycerol (TAG) precursors (Figs 1C and 2C). Collectively, these data suggest that FA synthesis is reduced in SCO2‐deficient vs proficient cells. However, in contrast to WT cells, numerous Nile Red‐positive lipid droplets (LD), which are depots of TAGs, were observed in *SCO2* ^−/−^ but not in WT HCT116 cells (Fig. 3D). SCO2‐deficient mice have increased fat mass associated with reduced β‐oxidation [38]. Altogether, these findings suggest that *SCO2* loss‐induced decrease in mitochondrial β‐oxidation is incompletely compensated by reduction in FA synthesis via suppression of ACC. Of note, we excluded the possibility that LDs contain FA that were imported from growth media by culturing cells in the presence of normal or FA‐free FBS for 20 days, which did not affect the size or number of LDs (Fig. S2A). Importantly, HCT116 WT cells phenocopied the accumulation of LDs observed in SCO2‐deficient cells when grown continuously under severe hypoxia (< 0.1% O_2_; Fig. S2B) or in the presence of complex III inhibitors antimycin A (Ant A) or myxothiazol (Myx; Fig. S2C). Chronically hypoxic HCT116 *SCO2* ^−/−^ cells also increased LD deposition, suggesting that in the presence of O_2_ some FA may be metabolized likely through the peroxisomal β‐oxidation [39].

### Complex IV dysfunction in HCT116 cells results in increased succinate and 2‐hydroxyglutarate levels

**Fig. 4:**
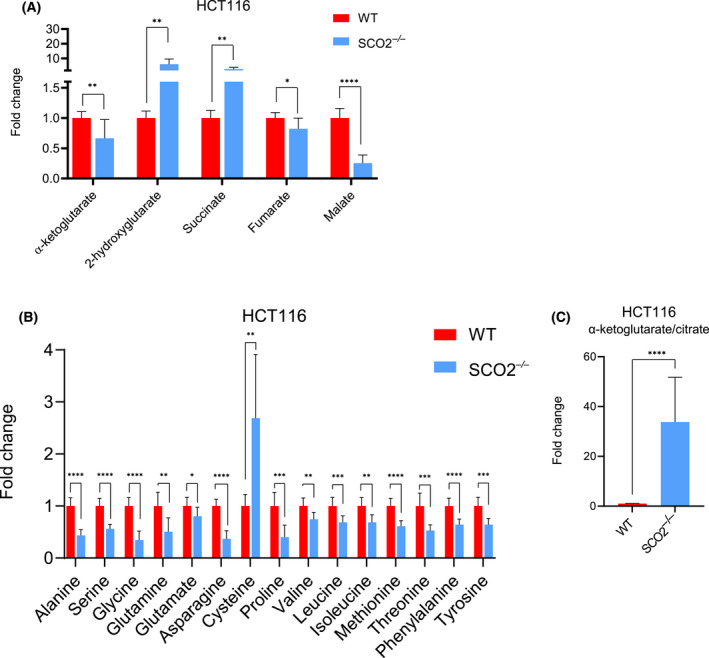
*SCO2* abrogation in HCT116 cells results in asymmetry in CAC intermediates and decrease in the levels of most amino acids. (A, B) Intracellular levels of metabolites in WT and *SCO2* ^−/−^ HCT116 cells were monitored by GC‐MS showing decreased levels of malate, fumarate, α‐ketoglutarate while succinate level increased in *SCO2* ^−/−^ HCT116 cells. Data are represented as mean fold change ± SD relative to WT HCT116 cells wherein values for each metabolite are set to 1. *P < 0.05, **P < 0.01, ***P < 0.001, ****P < 0.0001 (Unpaired two‐tailed *t*‐test; *n* = 3 independent experiments with three technical replicates in each). (C) Intracellular α‐ketoglutarate/citrate ratio in WT and *SCO2* ^−/−^ HCT116 cells was determined from the levels of respective metabolites detected by GC‐MS. The values obtained for WT HCT116 cells were set at 1 and the results were represented as means of the ratios from three independent experiments with three technical replicates each ± SD. ****P < 0.0001 (Unpaired two‐tailed *t*‐test).

Steady‐state metabolite analysis also revealed dramatic accumulation of succinate and 2‐hydroxyglutarate (2‐HG) in *SCO2* ^−/−^ vs WT HCT116 cells (Fig. 4A). As succinate is known to accumulate upon complex II inhibition [40, 41, 42, 43] as well as following treatment with the complex III inhibitor Ant A [44], we reasoned that the increased succinate observed in cells devoid of SCO2 was due to the impairment of the electron transport chain (ETC) and subsequent decrease in the activity of mitochondrial complex II (SDH). Indeed, steady‐state fumarate and malate levels were decreased, thus suggesting reduced utilization of succinate through CAC in *SCO2* ^−/−^ as compared to WT HCT116 cells (Fig. 4A). In addition, 2‐HG accumulated in HCT116 cells devoid of *SCO2*. While we could not distinguish the enantiomer of 2‐HG that was elevated in HCT116 *SCO2* ^−/−^ cells, sequencing analysis of *IDH1* and *IDH2* mRNAs in WT and HCT116 *SCO2* ^−/−^ did not reveal neomorphic mutations (arginine residues R100, R109, and R132 for IDH1, and R140, R149, and R172 for IDH2) known to give rise to D‐2‐HG (Fig. S3A) [45]. Low pH, reduced oxidation of α‐ketoglutarate (α‐KG), and increased NADH levels have been shown to enhance L‐2‐HG synthesis chiefly via lactate (LDH) and malate dehydrogenases (MDH) [46, 47]. Although we did not detect differences in cellular pH between WT and *SCO2* ^−/−^ HCT116 cells (Fig. S3B), the overall reductive state caused by ETC dysfunction and the lack of *IDH1* or *IDH2* mutations suggest that it is likely that L‐2‐HG accumulates in HCT116 *SCO2* ^−/−^ cells [48].

### *SCO2* loss in HCT116 cells affects amino acid metabolism

Amino acid metabolism is partly dependent on mitochondrial function. The CAC intermediates are used to biosynthesize amino acids including aspartate, asparagine, proline, and glutamate [49]. Moreover, as noted above, the essential role of mitochondrial respiration in producing aspartate to fuel neoplastic growth has been described [29, 30, 50]. To this end, it was shown that the inhibition of mitochondrial complex I with metformin not only reduced the NAD^+^/NADH ratio but also suppressed aspartate biosynthesis [50]. Consistently, we observed a decrease in proline, glutamate, and asparagine levels in *SCO2* ^−/−^ vs WT HCT116 cells (Fig. 4B). In turn, levels of cysteine were higher in SCO2‐deficient vs proficient HCT116 cells (Fig. 4B). Considering that cysteine is a rate‐limiting substrate in glutathione synthesis [51] and that the loss of *SCO2* results in increased ROS production [13], the observed increase in cysteine levels in *SCO2* ^−/−^ HCT116 cells may be indicative of potential compensatory mechanisms that are triggered to protect cells from ROS‐induced damage. In line with this, increased cysteine levels may be required to support the turnover of mitochondrial cysteine‐rich proteins that also contribute to the antioxidant defense machinery [52]. Accordingly, the levels of mitochondrial ROS scavenger superoxide dismutase 2 (SOD2) were elevated in *SCO2* ^−/−^ compared with WT HCT116 cells (Fig. S4A).

Cancer cells use glutamine as an anaplerotic source for the CAC [53, 54]. The loss of *SCO2* in HCT116 cells reduced intracellular glutamine levels compared with WT cells. Inhibition of OXPHOS has been shown to result in reductive glutamine metabolism and consequent reductive carboxylation of α‐KG to produce aspartate, which is necessary for cancer cell proliferation [29, 30]. A high α‐KG/citrate ratio, commonly observed in cancer cells with mitochondrial dysfunction, is an indirect indication of reductive glutamine metabolism [55]. Notwithstanding that the glutamine levels were lower in *SCO2* ^−/−^ as compared to WT HCT116 cells, the α‐KG/citrate ratio was dramatically elevated in SCO2‐deficient cells (Fig. 4B,C). Hence, although the deletion of *SCO2* decreased intracellular glutamine levels in HCT116 cells, these data suggest that most of the glutamine is likely to be reductively metabolized in HCT116 *SCO2* ^−/−^ cells. Altogether, these data suggest that the loss of SCO2 causes major perturbations in amino acid metabolism.

### Loss of mitochondrial complex IV function leads to induction of IGF1R/AKT axis with paradoxical reduction in mTOR signaling

**Fig. 5:**
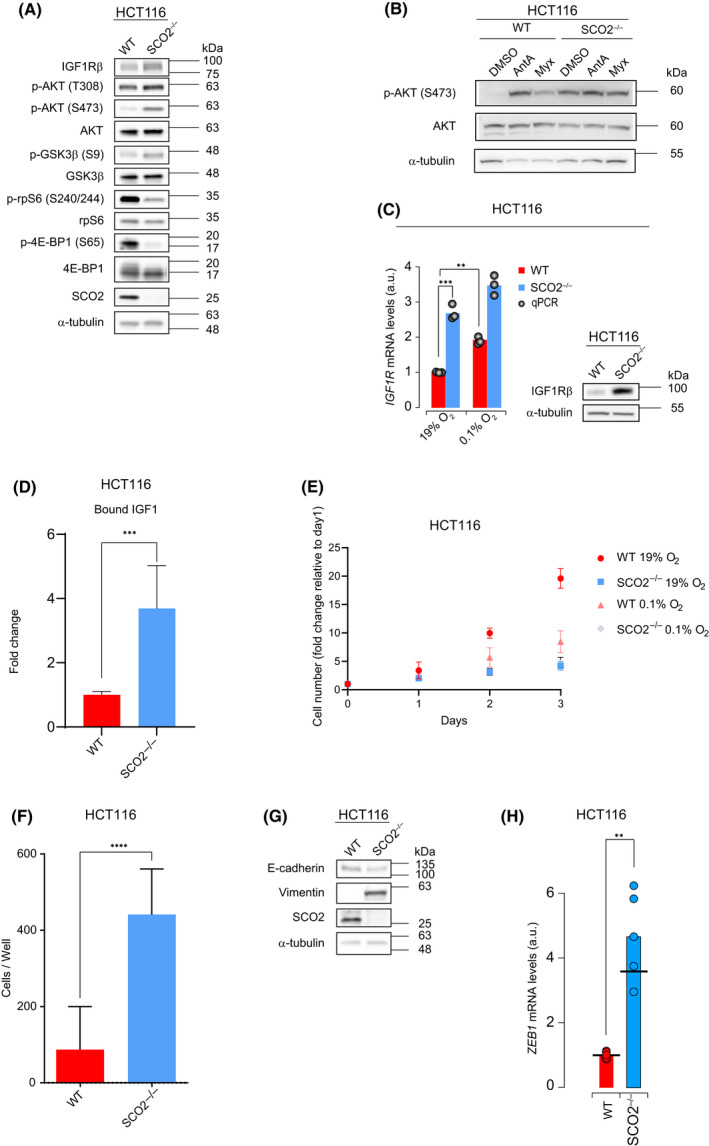
*SCO2* loss in HCT116 cells leads to activation of AKT and AMPK, suppression of mTORC1, reduction in cell proliferation and increased cell migration. (A) Levels and phosphorylation status of indicated proteins were monitored by Western blotting in WT and *SCO2* ^−/−^ HCT116 cells. α‐tubulin served as a loading control. Shown are representative Western blots from three independent replicates. (B) WT HCT116 cells were treated with antimycin A (Ant A, 5 µm) or myxothiazol (Myx, 2 µm) treated for 8 days. Western blot analysis was employed to assess the levels and phosphorylation status of indicated proteins. α‐tubulin served as a loading control. Representative blots from three independent experiments are shown (C). *IGF1R* mRNA and IGF1Rβ protein levels were determined by RT‐qPCR and Western blotting respectively. As indicated WT and *SCO2* ^−/−^ HCT116 cells were maintained under 19% or 0.1% O_2_ for 8 days. RT‐qPCR data are presented as mean values of three independent experiments (columns) and individual data points; mean values obtained for HCT116 WT maintained under 19% oxygen was set to 1 a.u. **P < 0.01, ***P < 0.001 (Unpaired two‐tailed *t*‐test). Representative Western blots from three independent experiments are shown. α‐tubulin was used as a loading control. (D) Levels of bound IGF1 were calculated by monitoring free IGF1 levels in extracellular media of WT and *SCO2* ^−/−^ HCT116 cells using ELISA. Data is represented as a mean fold change ± SD relative to WT HCT116 cells (set at 1). ****P < 0.0001 (Unpaired two‐tailed *t*‐test; *n* = 3 independent replicates with three technical replicates each). (E) Numbers of viable WT (red) and *SCO2* ^−/−^ (blue) HCT116 cells grown 0.1% (triangles) or 19% O_2_ (squares) for indicated times was monitored by trypan blue exclusion using automated cell counter. Results are presented as the mean value ± SD relative to WT or *SCO2* ^−/−^ HCT116 cell number grown in normoxia at day 1, which was set to 1 (*n* = 4 independent experiments with three technical replicates each). (F) Transwell migration assay of WT and *SCO2* ^−/−^ HCT116 cells. The columns represent the mean summarized cell counts of four fields from a single transwell migration chamber. Data are presented as the mean value ± SD ****P < 0.0001 (Unpaired two‐tailed *t*‐test; *n* = 3 independent experiments with two technical replicates each). (G) Levels of indicated proteins were monitored by Western blotting. α‐tubulin was used as a loading control. Representative blots from three independent experiments are shown. (H) *ZEB1* mRNA levels in WT and *SCO2* ^−/−^ HCT116 cells were analyzed by RT‐qPCR. Results are presented as mean values of three independent experiments (columns) and individual data points. Mean values obtained for WT HCT116 cells was set to 1 a.u. **P < 0.01, ***P < 0.001 (Unpaired two‐tailed *t*‐test). Quantifications of Western Blots are shown in Fig. S9.

To better understand the mechanism of metabolic adaptations to the disruption of mitochondrial complex IV function, we next investigated the effects of SCO2 abrogation on mTOR signaling, which acts as a major conductor of metabolic programs in the cell [56]. Notably, phosphorylation of AKT activation loop (T308) and hydrophobic motif (S473) was increased in *SCO2* ^−/−^ as compared to WT HCT116 cells (Fig. 5A). Accordingly, *SCO2* ^−/−^ HCT116 cells exhibited higher AKT‐dependent phosphorylation (S9) of GSK3β (Fig. 5A), thus indicating that AKT activity is higher in SCO2‐deficient vs proficient cells. Importantly, HCT116 WT cells treated with complex III inhibitors Ant A and Myx showed similar increase in phosphorylation of AKT (S473; Fig. 5B). Collectively, this indicates that disruption of terminal ETC complexes (III and IV) may result in compensatory AKT activation. Notably, the increase in AKT activity coincided with upregulation of *IGF1R* mRNA and IGF1Rβ protein in *SCO2* ^−/−^ vs WT HCT116 cells (Fig. 5A and 5C), implying that the loss of mitochondrial complex IV function may lead to increased signaling through the IGF1R/AKT axis. Similar induction of *IGF1R* mRNA levels was observed in WT HCT116 cells maintained at 0.1% O_2_ (Fig. 5C). Upregulated signaling via the IGF1R/AKT axis in *SCO2* ^−/−^ HCT116 cells was also paralleled by an increase in the amount of IGF1 bound to IGF1R (Fig. 5D).

Surprisingly, despite increased AKT activity, *SCO2* loss in HCT116 cells led to a decrease in mTORC1 activity, as illustrated by reduction in phosphorylation of its downstream substrate 4E‐binding protein 1 (4E‐BP1; S65), and ribosomal protein S6 (rpS6; S240/244), which is a substrate of mTOR‐dependent S6 kinases (S6Ks; Fig. 5A). In turn, AMPK phosphorylation was increased in *SCO2* ^−/−^ relative to HCT116 WT cells (Fig. 3B). Given that AMPK is a negative regulator of mTORC1 [57, 58], this may explain the reduction in mTORC1 signaling upon *SCO2* loss, despite increase in AKT activity. Altogether, these findings show that the impairment of mitochondrial complex IV function leads to increase in AKT and AMPK activity, which is accompanied by a decrease in mTORC1 signaling.

mTORC1 stimulates cell proliferation [59]. *SCO2* loss‐induced reduction in mTORC1 activity was therefore consistent with decreased proliferation of SCO2‐deficient vs proficient cells (Fig. 5E). Consistent with previous findings [13], the proliferation of *SCO2* ^−/−^ HCT116 cells was less affected by exposure to 0.1% O_2_ in comparison with WT HCT116 cells (Fig. 5E). These observed phenotypes appeared not to be limited to HCT116 cells. *SCO2* depletion in A549, HeLa, and HT29 cells resulted in increased AKT phosphorylation (S473) and decreased phosphorylation of downstream mTORC1 substrates (Fig. S5A‐C). Consistent with the findings in HCT116 cells, *SCO2* depletion also reduced the proliferation of A549, HeLa, and HT29 cells (Fig. S5D‐F). Next, we re‐expressed *SCO2* in *SCO2* ^−/−^ HCT116 cells to exclude potential inadvertent effects caused by cellular adaptation to *SCO2* loss. Notwithstanding that as indicated above exogenous SCO2 protein levels were significantly lower than the levels of endogenous SCO2 protein (Figs S1E and S5G), re‐expression of *SCO2* in *SCO2* ^−/−^ HCT116 cells resulted in partial rescue of cellular proliferation as compared to vector infected *SCO2* ^−/−^ HCT116 cells (Fig. S5H). Re‐expression of *SCO2* also partially rescued mTORC1 signaling as illustrated by an increase in 4E‐BP1 phosphorylation in *SCO2* ^−/−^ HCT116 cells expressing *SCO2* relative to control, vector infected *SCO2* ^−/−^ HCT116 cells (Fig. S5G).

### *SCO2* loss leads to increased cell migration and altered expression of EMT markers in HCT116 cells

Mitochondrial dysfunction resulting in increased ROS and/or accumulation of oncometabolites such as 2‐HG has been linked to elevated expression of EMT markers, increased cell migration, and higher metastatic potential of cancer cells [60, 61]. Accordingly, previous studies have shown higher levels of ROS and the mesenchymal marker, vimentin, in *SCO2* ^−/−^ compared with HCT116 WT cells [13, 14]. Based on this, we next monitored the effects of *SCO2* loss on migration and expression of epithelial (E‐cadherin) and mesenchymal (vimentin) markers using HCT116 cell model. These studies revealed that the loss of *SCO2* enhances the migration of HCT116 cells (Fig. 5F). In addition to increased vimentin levels, SCO2‐deficient HCT116 cells exhibited a decrease in E‐cadherin levels as compared to WT HCT116 cells (Fig. 5G). Also, the loss of *SCO2* in HCT116 cells correlated with elevated *ZEB1* mRNA levels (Fig. 5H), which encodes a key regulator of EMT [62]. Moreover, re‐expression of *SCO2* in *SCO2* ^−/−^ HCT116 cells attenuated migration, reduced vimentin levels while increasing E‐cadherin abundance (Fig. S5I,J). We observed that SCO2‐deficient HCT116 cells exhibit attenuated mTORC1 signaling as compared to their SCO2‐proficient counterparts (Fig. 5A). Inhibition of mTOR was shown to coincide with elevated phosphorylation of the α subunit of eukaryotic translation initiation factor 2 (eIF2) and increased cell migration [63, 64]. However, the phosphorylation of eIF2α (S51) was not affected by the SCO2 status in the HCT116 cells (Fig. S5K). Hence, although *SCO2* deletion in HCT116 cells reduced proliferation, it increased their migratory potential whereby this effect was independent of eIF2α phosphorylation.

### SCO2‐deficient HCT116 cells exhibit increased susceptibility to IGF1R and AKT inhibitors

**Fig. 6:**
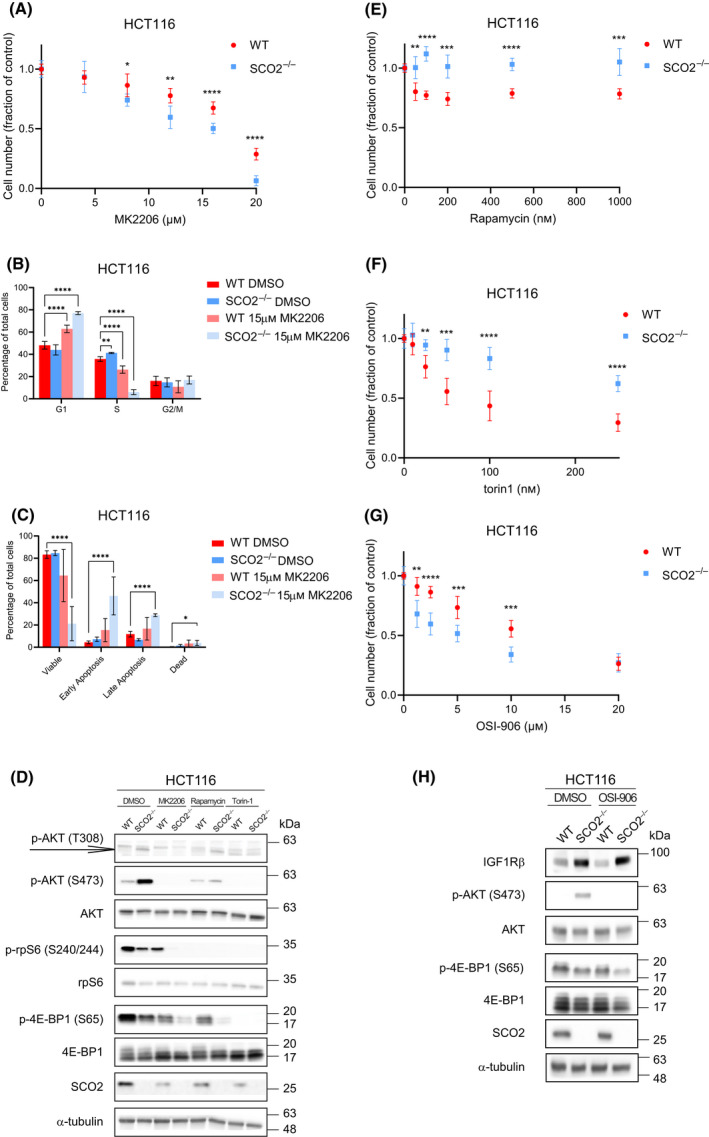
Complex IV deficiency increases sensitivity of HCT116 cells to IGF1R and AKT, but not mTOR inhibitors. (A) WT and *SCO2* ^−/−^ HCT116 cells were treated with indicated concentrations of pan‐AKT inhibitor MK2206 or a vehicle (DMSO) for 3 days. The number of viable cells at each time point was obtained using trypan blue exclusion and automated cell counter. Data are represented as the mean fraction of cells relative to the corresponding DMSO‐treated controls, which were set to 1. Experiments were carried out in three independent replicates (two technical replicates each time). Bars represent mean values ± SD values. (B) Cell cycle distribution of WT and *SCO2* ^−/−^ HCT116 cells treated with MK2206 (15 µm) or a vehicle (DMSO) for 24 h was determined by flow cytometry. The data are presented as mean percent values of the total cell population ± the SD. *P < 0.05, **P < 0.01, ***P < 0.001, ****P < 0.0001 (One‐way ANOVA; Dunnette's *post hoc* test with WT HCT116 DMSO‐treated cells as control; *n* = 2 independent experiments with three technical replicates in each). (C) WT and *SCO2* ^−/−^ HCT116 cells were treated with MK2206 (15 µm) for 48 h, stained with Annexin V‐FITC and PI subsequently monitored by flow cytometry. Percent of live, early apoptotic, late apoptotic and dead cells are shown relative to the total cell population. The data are presented as mean values ± the SD. *P < 0.05, **P < 0.01, ***P < 0.001, ****P < 0.0001 (One‐way ANOVA; Dunnette's *post hoc* test with WT HCT116 DMSO‐treated cells as control; *n* = 2 independent experiments with 3 technical replicates each). (D) Western blot analysis of indicated proteins isolated from WT and HCT116 *SCO2* ^−/−^ cells treated with MK2206 (15 µm), rapamycin (50 nm), torin 1 (250 nm), or a vehicle (DMSO) for 24 h. Representative blots from two independent experiments are shown. α‐tubulin served as a loading control. (E–G), WT and *SCO2* ^−/−^ HCT116 cells were treated with indicated concentrations of mTOR [rapamycin (E) or torin 1 (F)], INSR/IGF1R inhibitors [OSI‐906 (G)] or a vehicle (DMSO) for 3 days. Numbers of viable cells in each condition were determined by trypan blue exclusion using automated cell counter. Data are represented as the mean fraction of cells relative to DMSO‐treated controls ± SD. Experiments were carried out in independent triplicate with two technical replicates each. (H) WT and *SCO2* ^−/−^ HCT116 cells were treated with OS1‐906 (10 µm) or a vehicle (DMSO) for 24 h. Levels and phosphorylation status of indicated proteins were determined by Western blotting. α‐tubulin served as a loading control. Representative blots from 3 independent experiments are shown. Quantifications of Western Blots are shown in Fig. S9.

Considering that the disruption of mitochondrial complex IV activity is paralleled by the increase in IGF1Rβ levels and AKT activity (Fig. 5A), we next investigated the effects of IGF1R and AKT inhibitors on the fate of HCT116 cells as a function of their SCO2 status. As compared to control (vehicle; DMSO) treatment, allosteric pan‐AKT inhibitor MK2206 reduced proliferation (Fig. 6A), attenuated progression from G1 to S phase of cell cycle (Fig. 6B), and decreased survival (Fig. 6C) of *SCO2* ^−/−^ HCT116 cells more pronouncedly than in WT cells. Of note, depletion of SCO2 also potentiated anti‐proliferative effects of MK2206 in A549 and HT29 cells as compared to the control, scrambled shRNA infected cells (Fig. S6A,B) albeit to a lesser extent than the complete SCO2 knock‐out in HCT116 cells (Fig. 6A). Moreover, MK2206 reduced levels of glycolytic intermediates including DHAP and 3‐PG in HCT116 *SCO2* ^−/−^ cells to a greater extent than in control cells (Fig. S7A,B). Notably, comparable reduction in phosphorylated AKT levels was observed between WT and *SCO2* ^−/−^ HCT116 cells, thus excluding potential differences in AKT inhibition by MK2206 between these cell lines (Fig. 6D). The effect of MK2206 on mTORC1, as monitored by rpS6 (S240/244) and 4E‐BP1 (S65) phosphorylation levels, was greater in SCO2‐proficient vs deficient HCT116 cells, which is consistent with higher basal mTORC1 activity in the former cell line (Fig. 6D). In turn, although the allosteric (rapamycin) and active‐site (torin 1) mTOR inhibitors abolished mTORC1 signaling in both cell lines as evidenced by the reduction in rpS6 (S240/244) and 4E‐BP1 (S65) phosphorylation, their anti‐proliferative effects were lesser in *SCO2* ^−/−^ as compared to WT HCT116 cells (Fig.6D‐F). SCO2 depletion also attenuated anti‐proliferative effects of mTOR inhibitors in A549 and HT29 cells (Fig. S8A–D). Similar to AKT inhibition, IGF1R/insulin receptor inhibitor, OSI‐906 (linsitinib), reduced 4E‐BP1 (S65) phosphorylation, and proliferation of HCT116 *SCO2* ^−/−^ cells to a greater extent than in WT cells (Fig. 6G,H). These findings suggest that the loss of *SCO2* may render HCT116 cells “addicted” to the IGF1R/AKT axis. In turn, HCT116 *SCO2* ^−/−^ cells exhibit reduced mTORC1 signaling, which may explain their decreased sensitivity to mTOR inhibitors. Collectively, these results suggest that uncoupling of IGF1R/AKT and mTORC1 signaling may be required for the adaptation of HCT116 cells to *SCO2* loss. mTOR is major stimulator of anabolic processes such as lipid and protein synthesis [65, 66]. This alludes to a model whereby activation of IGF1R/AKT axis promotes survival of cancer cells with complex IV dysfunction by driving glycolysis, while concomitant suppression of mTOR is required to decrease energy consumption and thus compensate for disrupted mitochondrial ATP production.

## Discussion

Metabolic reprogramming of cancer cells allows them to adapt to changes in nutrient and oxygen availability and therapeutic insults [67, 68]. To this end, it is considered that metabolic rewiring not only fulfills the energetic requirements of cancer cells, but also provides building blocks and maintains redox buffering capacity required to fuel the growth and survival of cancer cells [67, 68]. Herein, we report that disruption of mitochondrial complex IV function by *SCO2* loss is compensated by dramatic perturbations in metabolome and signaling. Most notably, *SCO2* loss in HCT116 cells leads to increase in glycolysis [12]. This not only enables HCT116 cells with dysfunctional complex IV to meet the biosynthetic demands by providing carbon atoms but also allows for the regeneration of NAD^+^ which is necessary to maintain glycolysis and redox homeostasis. Indeed, although *SCO2* loss leads to overall increase in NADH levels in HCT116 cells [13], *SCO2* ^−/−^ HCT116 cells upregulate NAD^+^ regenerating reactions. This is consistent with previous findings suggesting that conversion of pyruvate to lactate and the simultaneous production of NAD^+^ from NADH by lactate dehydrogenase are essential in the context of mitochondrial dysfunction [69]. Moreover, *SCO2* ^−/−^ HCT116 cells appear to exhibit increased activity of GPD1, as illustrated by the alterations in steady‐state metabolite levels and increased glucose flux to 3‐PG. Notably, although mitochondrial glycerophosphate dehydrogenase (GPD2) has been implicated in the reoxidation of cytosolic NADH via the ETC in prostate cancer [70], due to compromised mitochondrial functions, this mechanism is unlikely to be at play in SCO2‐deficient HCT116 cells. Hence, GPD1 is most likely involved in maintaining NAD^+^/NADH ratio in HCT116 *SCO2* ^−/−^ cells. In addition, we observed large accumulation of lipids in HCT116 *SCO2* ^−/−^ cells as compared to their WT counterparts. Lipid accumulation in SCO2‐deficient cells was not caused by increased lipogenesis or FA uptake, thus indicating that the observed phenotype likely stems from impediments in β‐oxidation of FA in the mitochondria. Finally, *SCO2* loss resulted in a reduction of most of the CAC intermediates and amino acids, which was paralleled by an increase in succinate and 2‐HG. Although we did not formally test whether accumulation of these metabolites is sufficient to disrupt the function of α‐ketoglutarate‐dependent enzymes (e.g., HIF‐α prolyl hydroxylase, histone, DNA, and RNA demethylases) [41, 67], these findings suggest that *SCO2* loss may result in epigenetic, transcriptional, and epitranscriptional reprogramming.

Notably, the observed alterations in metabolic programs in SCO2‐deficient HCT116 cells were paralleled by significant rewiring characterized by increased IGF1R/AKT activity that was accompanied by a reduction in mTORC1 signaling. This, at least in part, may be explained by elevated AMPK activity in SCO2‐deficient HCT116 cells. Accordingly, *SCO2* ^−/−^ HCT116 cells showed heightened sensitivity to IGF1R and AKT inhibitors, while they were less sensitive to mTOR inhibitors relative to SCO2‐proficient cells. Previous studies demonstrated that AKT upregulates glycolysis [71]. Since the loss of *SCO2* renders HCT116 cells heavily dependent on glycolysis to produce ATP and regenerate NAD^+^, it is plausible that these cells are more sensitive to IGF1R and AKT inhibitors because of their inhibitory effects on glycolysis. Strikingly, similar induction in AKT phosphorylation was observed in WT HCT116 cells treated with mitochondrial complex III inhibitors or subjected to hypoxia. Comparable to HCT116 *SCO2* ^−/−^ cells, the decreased expression of *SCO2* in HeLa and A549 increased AKT activity while downregulating mTORC1. Although the precise mechanism(s) of induction of IGF1R/AKT axis in the context of mitochondrial complex III or IV disruption remains elusive, these findings suggest that mitochondrial dysfunction may lead to critical reliance of neoplastic cells on AKT.

AMPK acts as a negative regulator of mTORC1, and it has recently been described that it may stimulate mTORC2 [72] and be activated by ROS [73]. This suggests that AMPK upregulation in HCT116 *SCO2* ^−/−^ cells may dampen mTORC1 signaling while sustaining mTORC2 and AKT activity. Indeed, we observed that disruption of *SCO2* results in marked increase in S473 AKT phosphorylation, which is indicative of increased mTORC2 activity [74]. In addition, mTORC2 may also exert AKT‐independent effects of glucose metabolism as reported in glioblastoma [75]. Of note, HCT116 *SCO2* ^−/−^ cells produce ATP mainly in cytosol and have the ATP/protein ratio similar to that in WT cells [14], thus suggesting that AMP/ATP or ADP/ATP ratio may not play a major role in AMPK activation in this context. In turn, cytosolic Ca^2+^ levels are elevated in *SCO2* ^−/−^ as compared to HCT116 WT cells [14], thereby suggesting that Ca^2+^/calmodulin‐dependent protein kinase kinase (CaMKK) may activate AMPK in cells exhibiting complex IV dysfunction. Also, AMPK has been found to be hyperactivated by CaMKK in mouse embryonic fibroblasts (MEFs) devoid of LKB1 kinase when subjected to oxidative stress [76]. Considering that we observed similar phenotypes between cells with functional (HCT116) or dysfunctional LKB1 (A549, HeLa), it is likely that mechanisms other than LKB1 play a major role in AMPK activation in the context of mitochondrial complex IV dysfunction. Intriguingly, despite several attempts, we failed to knock down AMPK α1 and/or α2 subunits in HCT116 *SCO2* ^−/−^ cells, or deplete SCO2 in AMPK α1/α2^−/−^ MEFs due to massive cell death. Notwithstanding that future investigations are required to establish the precise mechanism(s) of adaptation of cancer cells to the abrogated complex IV activity, these observations may suggest an important role for AMPK in this process.

Recent studies show that ROS plays pivotal roles in facilitating EMT and cell motility [77]. HCT116 *SCO2* ^−/−^ cells were previously shown to have elevated ROS levels [13]. A prior study showed that ZEB1 represses E‐cadherin and induces vimentin [62]. Furthermore, elevated levels of 2‐HG have been reported to increase the expression of *ZEB1* by inhibiting histone demethylase, which in turn decreased the expression of E‐cadherin while concurrently increasing the expression of vimentin in HCT116 cells [78]. Similarly, elevated levels of 2‐HG were observed to stimulate migration and invasion of HCT116 cells and associated with distant metastasis [78]. Collectively, these findings suggest that metabolic and signaling adaptation to mitochondrial dysfunction caused by *SCO2* loss result in increased migratory potential of HCT116 cells.

Since *SCO2* represents one of the major metabolic targets of p53, together with the previous work, our study suggests that complex IV dysfunction plays a major role in metabolic perturbations of malignancies with defective p53 function. Importantly, we demonstrate that despite reducing proliferation in cell culture models, *SCO2* loss results in alterations in expression of EMT markers and increased migration of HCT116 cells. Considering the positive correlation of these phenomena and metastatic spread of cancer [79, 80], these findings suggest that alterations in *SCO2* and/or dysfunction of mitochondrial complex IV may increase the metastatic potential of cancer cells. Nonetheless, we acknowledge that our study has significant limitations as it was carried out on a very limited number of cell lines, while the precise mechanisms of signaling and metabolic perturbations remain elusive. Expanding these studies across various cancer cell lines and *in vivo* is therefore warranted to establish the general role of SCO2 and disruption of complex IV function in metabolic reprogramming and accompanied signaling rewiring in neoplasia and its impact on cancer cell phenotypes.

## Methods

### Cell lines and culture conditions

*SCO2* ^−/−^ and WT HCT116 cells were provided by P. M. Hwang [15]. A549, HT29, and HeLa cells were obtained and authenticated by ATCC (10801 University Blvd, Manassas, Virginia, USA). All cells were cultured in McCoy's 5A medium supplemented with 10% heat‐inactivated FBS, 1% penicillin/streptomycin, 10 mm HEPES and 2 mm l‐Glutamine (provided by either MilliporeSigma or Wisent, ST‐BRUNO, Quebec J3V 4P8 Canada). Cells were maintained at 37 °C, 5% CO_2_ and 19% O_2_, unless otherwise indicated.

### Lentiviral infection of *SCO2* shRNA and expression constructs

Stable knockdown of *SCO2* in A549, HT29, and HeLa cells was generated using pLKO.1 lentiviral shSCO2‐containing vector (TRCN236560) obtained from Mission TRC genome‐wide shRNA collections (MilliporeSigma). Non‐mammalian shRNA control plasmid DNA was used as a negative control (MilliporeSigma: SHC002).

For *SCO2* cDNA generation, RNA was extracted from HCT116 cells using Trizol (Thermo Fisher Scientific, 3410 Griffith St, Saint‐Laurent, Quebec H4T 1Y6, Canada) according to the manufacturer's instructions. Subsequently, cDNA was synthesized by SuperScript III Reverse Transcriptase (Invitrogen, Thermo Fisher Scientific, 3410 Griffith St, Saint‐Laurent, Quebec H4T 1Y6, Canada) and PCR amplified using Phusion High‐Fidelity DNA polymerase (New England BioLabs, 9 Carlow Ct, Whitby, Ontario L1N 9T7, Canada) and the following primers:

Forward primer: 5′ TATGCAACCGGTATGTTTGGTGGAGGTGGAGTTCTGAGC 3′

Reverse Primer: 5′ TTGCAAGAATTCTCAAGACAGGACACTGCGGAAAGCCGC 3′

PCR cycling conditions: Initial denaturation was done at 98 °C for 3 min, 35 cycles of 98 °C for 30 s, 60 °C for 30 s, and 72 °C for 40 s, and the final extension was done at 72 °C for 10 min.

Human *SCO2* cDNA was cloned into pLKO.1‐puro empty vector control plasmid DNA (SHC001) using EcoRI and AgeI restriction enzymes (New England BioLabs). pLKO.1‐puro empty vector (SHC001) was used as a negative control for the exogenous expression of *SCO2*.

Lentiviruses were produced as follows: HEK293T cells were co‐transfected with 12 µg of shSCO2‐containing plasmid (to knockdown *SCO2* in HeLa, HT29 and A549 cells) or *SCO2*‐containing plasmid (to re‐express *SCO2* in HCT116 *SCO2* ^−/−^ cells), 8 µg of psPAX2 packaging plasmid, and 4 µg of pMD2.G plasmid using jetPRIME transfection agent according to the manufacturer's protocol (Polyplus transfection, VECTURA, 75 Rue Marguerite Perey, 67400 Illkirch‐Graffenstaden, France). The media were changed 24 h later and collected 48 h post‐transfection. The media were filtered through 0.45 µm filter (Fisher Scientific). The virus‐containing media were mixed with fresh media at 1 : 1 ratio and added to already seeded A549, HT29, and HeLa cells. 4 µg·mL^−1^ polybrene (MilliporeSigma) was added to the cells. Cells were re‐infected the following day using freshly collected and filtered virus‐containing media. 48 h later, selection was done using 2 µg·mL^−1^ of puromycin (Bio Basic, 20 Konrad Crescent, Markham, Ontario L3R 8T4, Canada).

### Flow cytometry analysis of cell cycle

HCT116 *SCO2* ^−/−^ and WT cells were seeded on 6‐well plates and treated for 24 h MK2206 (15 µm) or vehicle (DMSO). The cells were then trypsinized, washed in PBS (Wisent), and then stained with 50 μg·mL^−1^ propidium iodide (PI) in cold hypotonic buffer (0.1% sodium citrate and 0.1% Triton X‐100 in water). Approximately, 2.5 × 10^5^ cells per condition were stained with 0.5 mL of the PI stain and analyzed on a LSR Fortessa cytometer (Becton Dickinson, 2100 Derry Rd W Suite 100 Mississauga, Ontario, L5N 0B3 Canada). Fluorescence was detected by excitation at 561 nm and acquisition on the 610/20‐A channel in linear scale. The distribution of cell cycle population was analyzed using the modfit lt (Verity Software House, Topsham, ME, USA) software.

### Flow cytometry analysis of apoptosis

HCT116 *SCO2* ^−/−^ and WT cells were seeded on six‐well plates and treated for 48h with MK2206 (15 µm) or vehicle (DMSO). Cells were trypsinized and counted, and 1 × 10^5^ cells were stained using Annexin V‐FITC and PI for 20 min in the dark as per the manufacturer's instructions (FITC‐Annexin V Apoptosis Detection Kit; Becton Dickinson). Samples were then analyzed with a LSR Fortessa cytometer (Becton Dickinson). Fluorescence was detected by excitation at 488 nm and acquisition on the 530/30‐A channel for FITC‐Annexin V and by excitation at 561 nm and acquisition on the 610/20‐A channel for PI. Cell populations were separated as follows: viable cells—Annexin V^−^/PI^−^; early apoptosis—Annexin V^+^/PI^−^; late apoptosis Annexin V^+^/PI^+^; dead Annexin V^−^/PI^+^; and expressed as % of total single cells.

### GC‐MS for stable isotope tracer analysis

3 × 10^5^ *SCO2* ^−/−^ and WT HCT116 cells were seeded on 6‐well plates to obtain 80% confluency 24 h later. For stable isotope tracer analysis (SITA), the supplemented McCoy's 5A medium was aspirated and replaced with fresh Dulbecco's Modified Eagle's Medium (DMEM) without glucose, glutamine, and pyruvate, supplemented with 10% heat‐inactivated FBS, 1% P/S, 2 mm l‐glutamine and 16.65 mm glucose to equilibrate the cells for 2 h. The equilibration media were replaced with DMEM containing 16.65 mm [U–^13^C]‐glucose (Cambridge Isotope laboratories, 331 Rue Deslauriers, Saint‐Laurent, Quebec H4N 1W2, Canada). Cells were incubated in this “labeling media” for 1 min, 2 min, 5 min, 10 min, 20 min, 30 min, 1 h, 2 h, and 4.5 h. The six‐well plates were taken out of the incubator and placed immediately on ice. The labeling media were aspirated, and cells were washed 3X with pre‐chilled isotonic saline solution (on ice) and quenched on dry ice by adding 600 µL of 80% methanol pre‐chilled to −20 °C. Cells were scraped from the plates and transferred to microcentrifuge tubes pre‐chilled to −20 °C. Cell suspensions were lysed using a sonicator at 4 °C (10 min, 30 s on, 30 s off, high power setting with a Diagenode Bioruptor, Diagenode Inc., Denville, New Jersey, United States). This was repeated to ensure complete recovery of metabolites. Cell debris was discarded after centrifugation (16,000 ***g***, 4 °C), and supernatants were transferred to pre‐chilled tubes and dried in a CentriVap cold trap (Labconco) overnight at 4 °C. Dried pellets were dissolved in 30 µL of pyridine containing methoxyamine‐HCl (10 mg·mL^−1^) (MilliporeSigma) using a sonicator and vortex. Samples were incubated for 30 min at 70 °C and then transferred to GC‐MS injection vials containing 70 µL of *N*‐tert‐Butyldimethylsilyl‐*N*‐methyltrifluoroacetamide (MTBSTFA). Sample mixtures were further incubated at 70 °C for 1 h. One microliter of each sample was injected for GC–MS analysis. GC–MS instrumentation and software were all from Agilent. GC–MS methods and mass isotopomer distribution analyses were conducted as described [81]. Data analyses were performed using the agilent chemstation and masshunter software (Agilent, boulevard 2c9, 2250 Bd Alfred Nobel, Saint‐Laurent, Quebec H4S 2C9 Canada).

### GC‐MS for Steady‐State metabolite analysis

3 × 10^5^ HCT116 *SCO2* ^−/−^ and WT cells were seeded on six‐well plates to obtain cells at 80% confluency 24 h later. The plates were quickly placed on ice after incubation period was over. Media were aspirated, and cells were washed 3X with chilled isotonic saline solution. Subsequently, 300 µL of 80% methanol pre‐chilled to −20 °C was added to the cells. Cells were scraped from the wells and transferred to microcentrifuge tubes pre‐chilled to −20 °C. 300 µL more of the 80% methanol was added to the leftover cells in the wells, scraped, collected, and pooled with the previously collected 300 µL fraction. Cells were lysed, centrifuged, and then the supernatants transferred into pre‐chilled microcentrifuge tubes as previously described for SITA. The collected supernatants were spiked with 750 ng of myristic acid‐D_27_ (Millipore Sigma, 2149 Winston Park Dr, Oakville, Ontario L6H 6J8, Canada) to serve as an internal standard. Supernatants were dried overnight and derivatized as previously described for SITA. One microliter of the derivatized samples was injected for GC–MS analysis. The instrumentation and software used were identical to those used for SITA. Each metabolite was normalized to the peak intensity of myristic acid‐D_27_ and the average protein content derived from cells seeded in parallel and identical conditions to those collected for GC‐MS steady‐state analysis. Data were expressed as fold change relative to WT HCT116 cells.

### Glucose and lactate release assays

5 × 10^5^ cells were seeded in triplicates in six‐well plates in 2 mL of media. The media were collected 24 h later. Cells were trypsinized and counted using an automated cell counter (Invitrogen). Media were spun down at 16,000 ***g*** for 10 min and the supernatants transferred into microcentrifuge tubes. Glucose and lactate concentrations in the media were measured using a BioProfile 400 analyzer (Nova Biomedical, 2900 Argentia Rd #17, Mississauga, Ontario L5N 7X9, Canada). Total uptake and release were calculated by subtracting the concentrations from baseline glucose and lactate concentrations measured in samples of media incubated under identical conditions in six‐well plates without cells. Molar concentrations of the metabolites were normalized per cell and presented relative to the control.

### Cell proliferation assays

1 × 10^5^ cells were seeded in 6‐well plates and incubated for 24 h. The media were replaced with treatment media containing MK2206, OSI‐906, rapamycin, torin 1, or DMSO as a negative control and incubated for 72 h. Treatment media were aspirated, and the cells were trypsinized. Complete media were added to stop the trypsinization. Samples were collected, stained with trypan blue to exclude dead cells, and counted using an automated cell counter (Invitrogen). For hypoxic treatments, 1 × 10^5^ cells were seeded in six‐well plates and placed in hypoxic chamber set to 0.1% O_2_. Cells were counted daily over a span of 3 days in parallel to cells seeded and placed at 19% O_2_.

### Western blotting and antibodies

Cells were washed with ice‐cold PBS and lysed for 20 min on ice in buffer A (Pierce RIPA, Thermo Fisher Scientific, 3410 Griffith St, Saint‐Laurent, Quebec H4T 1Y6, Canada) or B [50 mm Tris/HCL (pH 7.4), 5 mm NaF, 5 mm Na pyrophosphate, 1 mm EDTA, 1 mm EGTA, 250 mm mannitol, 1% (v/v) triton X‐100, 1 mm DTT], both supplemented with 1× complete protease inhibitors and 1× PhosSTOP. The lysates were clarified at 4 °C (10 min at 16,000 ***g***), and protein concentrations in the supernatants were determined using BCA™ (Pierce, Thermo Fisher Scientific, 3410 Griffith St, Saint‐Laurent, Quebec H4T 1Y6, Canada) kit. Samples were boiled in 1× Laemmli buffer at 95 °C for 5 min, proteins were separated by SDS/PAGE (10–40 μg per lane) and transferred using wet mini‐transfer system Hoefer™ TE 22 (Hoefer, CA, USA) either onto 0.45 μm Immobilon™‐P (Sigma‐Aldrich, 2149 Winston Park Dr, Oakville, Ontario L6H 6J8 Canada) poly(vinylidene difluoride) membranes (buffer A) or 0.45 μm nitrocellulose membranes (buffer B). In most cases, membranes were blocked in 5% BSA w/v in TBST buffer (0.1% Tween 20 in 1× TBS) for 1h and then incubated with primary antibodies, which were prepared at 1 : 1000 dilution in 5% BSA in TBST (16 h at 4 °C). Antibodies against α‐tubulin and HIF‐2α (1 : 1000 dilution) and the corresponding blocking solution were prepared using 5% (w/v) non‐fat dry milk in TBST. Membranes were washed with TBST (3–5 × 5–10 min) and incubated for 1 h with HRP‐conjugated secondary antibodies, which were prepared at 1 : 5000 or 1 : 2500 in 5% milk/TBST. Primary antibodies against 4E‐BP1#9644, p‐4E‐BP1 (S65) #9456, ACC #3662, anti p‐ACC (S79) #3661, eIF2α #2103S, p‐eIF2α (S51) #9721S, α‐tubulin #2125, acetyl‐α‐tubulin (Lys40) #5335, AKT #4691, p‐AKT (S473) #4060, p‐AKT (T308) #13038, #4056 and #9275, AMPKα #5832 and 2532, p‐AMPKα (T172) #50081 and # 2535, E‐cadherin #3195, IGF1Rβ #9750, mTOR #2972, p‐mTOR (Ser2448) #2971, rpS6 #2217, p‐rpS6 (S240/244) #2215, Snail #3879, Vimentin #5741 were all from Cell Signaling Technologies (Danvers, MA, USA); VDAC1 #sc‐8828 and rpS6 #sc‐74459 from Santa Cruz Biotechnologies (Dallas, TX, USA); SCO2 #PA5‐76209 from Thermo Fisher Scientific, HIF‐2α # AF2886 from R&D Systems (Minneapolis, MN, USA), SOD2 #ab16956, PDH E1‐α # Ab110330 and p‐PDH E1‐α (Ser293) #Ab92696 from Abcam (Cambridge, UK), acetyl‐lysine #ST1027 and α‐tubulin #T5168 from MilliporeSigma. Secondary HRP‐conjugated mouse anti‐goat/sheep IgG #A9452, mouse anti‐rabbit IgG #A1949, and goat anti‐mouse IgG #A0168 were from MilliporeSigma. After washing the membranes with TBST (3–5 × 5–10 min), specific protein bands were revealed by chemiluminescence using ECL™ (Pierce, Thermo Fisher Scientific, 3410 Griffith St, Saint‐Laurent, Quebec H4T 1Y6, Canada) Prime reagent on the LAS‐3000 imager (Fujifilm, 600 Suffolk Ct, Mississauga, Ontario L5R 4G4, Canada). As requested by reviewers, we performed densitometric analysis using imagej software. Herein, for each replicate intensities obtained for the bands of the proteins or phosphoproteins of interest were normalized against corresponding loading controls and total proteins, respectively. Resulting quantification (mean values across replicates ± standard deviation) is provided in Fig. S9, while X‐ray film scans and images of the immunoblots are included in Fig. S10.

### Transwell migration assay

Cells were serum starved overnight (16 h) and plated onto 12‐well‐transwell migration inserts of 8 µm pore diameter (VWR) at 2.5 × 10^5^ cells per well. 10% FBS‐supplemented media were added to the lower chamber as the chemo‐attractant and incubated for 24 h. Cells were fixed with 10% buffered formalin and stained with crystal violet. Migrated cells were imaged under 200X light microscope. Four separate images (fields) were taken of each well. Each data point represents the summarized cell count of four fields from a single transwell migration chamber.

### Generation of mRNA‐seq libraries and *IDH1* and *2* mRNA sequencing analysis

The libraries for mRNA sequencing analysis were generated as in [82]. Libraries were sequenced on an Illumina HiSeq 2000 system at the Beijing Genomics Institute (Building NO.7, BGI Park, No.21 Hongan 3rd Street, Yantian District, Shenzhen 518083, China).

### Isolation of RNA and RT‐PCR analysis

Isolation of total RNA and reverse transcription reaction were performed using RNeasy® plus universal mini kit (Qiagen, 81 Bay Street, Suite 4400. Toronto, Ontario M5J 2T3, Canada), high‐capacity cDNA reverse transcription kit (Applied Biosystems, Thermo Fisher Scientific, 3410 Griffith St, Saint‐Laurent, Quebec H4T 1Y6, Canada). qPCR was conducted using SensiFAST™ SYBR^®^ Lo‐ROX kit (Bioline, 3971 Old Walnut Rd, Alvinston, Ontario N0N 1A0, Canada) on the AB7300 machine and analyzed using the 7300 system sds Software (Applied Biosystems); reaction was controlled for the absence of genomic DNA amplification. Each experiment was carried out in independent triplicate. Primers were designed using Primer‐BLAST program (http://www.ncbi.nlm.nih.gov/tools/primer‐blast/) for human genes encoding β‐actin (NM_001101.5, forward: 5′‐CGGCTACAGCTTCACCACCACG and reverse: 5′‐AGGCTGGAAGAGTGCCTCAGGG) and IGF1R (NM_000875.5, forward: 5′‐CGGGGAGAGAGCCTCCTGTGA and reverse: 5′‐GCTGTTGGAGCCGCAGGCAT) and ZEB1 (NM_001128128.2, forward: 5′‐GAAGACAAACTGCATATTGTGGAAG and reverse: 5′‐CATCCTGCTTCATCTGCCTGA).

### Live cell staining and confocal microscopy

Loading of the cells with fluorescent indicators was performed for 30 min using 1 µm BCECF (whole cell pH probe) or 2 µg·mL^−1^ Nile Red (NR, lipid droplet stain) prepared in OptiMEM I medium. Live cell imaging was performed as follows:

Fluorescence lifetime imaging (FLIM) of BCECF was performed at 37 °C on an upright laser scanning Axio Examiner Z1 (Carl Zeiss, 1375 Trans Canada Route, Dorval, Quebec H9P 2V3, Canada) microscope equipped with 20×/1.0 W‐Plan Apochromat dipping objective. Fluorescence decays were collected using a picosecond 488 nm laser (with emission collected at 512–536 nm), DCS‐120 confocal time‐correlated single photon counting (TCSPC) scanner, photon counting detector and spcm software (Becker & Hickl GmbH, Nunsdorfer Ring 7 ‐ 9, 12277 Berlin, Germany). Data were analyzed with SPCImage (B&H) and Excel software. Fluorescence lifetime (LT) of BCECF was calculated using monoexponential fitting. The LT distribution histograms were obtained for three individual focal planes within each field of view (256 × 256‐pixel matrixes). Cumulative LT distribution histograms were produced according to the algorithm developed in [83].

The NR staining was analyzed on an Olympus (25 Leek Crescent, Richmond Hill, ON L4B 4B3, Canada) FV1000 confocal laser scanning microscope with controlled CO_2_, humidity, and temperature. Fluorescence signals were collected with a UPLSAPO 60X/1.35 oil immersion Super Apochromat objective using 543 nm excitation and 550–650 nm emission wavelengths. The resulting z‐stacked fluorescence and single‐plane differential interference contrast (DIC) images were processed using fv1000 viewer software (Olympus) and Adobe Photoshop (343 Preston St, Ottawa, ON K1S 1N4, Canada).

### IGF1 ELISA

1 × 10^6^ *SCO2* ^−/−^ and WT HCT116 cells were seeded in duplicate in 2 mL of media in six‐well plates and incubated for 24 h. The media were collected 24 h later, and the cells were trypsinized and counted using an automated cell counter (Invitrogen). Media samples were spun down at 16,000 ***g*** for 10 min, and the supernatants were transferred into microcentrifuge tubes. ELISA was used to measure the concentrations of free IGF1 present in the media samples (Ansh Labs, 445 W. Medical Center Blvd, Webster, Texas 77598, United States) according to the manufacturer's instructions. Briefly, media samples were added to IGF1 antibody‐coated microtiter wells. The wells were then washed and then incubated with horseradish peroxidase‐labeled antibody conjugate. At the end of the incubation period, a substrate solution was added to the wells until the color was adequately developed. After incubation with the substrate solution, an acidic stopping solution was added. Dual wavelength absorbance measurements were taken at 450 nm and at 630 nm. IGF1 concentrations were determined using a calibration curve. IGF1 bound to the cells was calculated by subtracting the concentrations of free IGF1 concentrations from baseline concentrations measured in samples of media incubated under identical conditions in 6‐well plates without cells. Molar concentrations of the metabolites were normalized per cell and presented relative to the control.

### Statistical analysis

Statistical analysis was performed using the results of 2–5 independent experiments. To ensure the accuracy and fidelity of the data, the experiments, when possible, were performed in several technical replicates.

The differences between *SCO2* ^−/−^ and WT HCT116 cells (at different treatment conditions) in NR fluorescence, protein and mRNA levels, and other measured parameters were evaluated using unpaired two‐tailed *t*‐test and non‐parametrical Mann–Whitney *U*‐test. Unpaired two‐tailed *t*‐tests, two‐way ANOVA (Dunnette's multiple comparison *post hoc* test), and one‐way ANOVA (Tukey's and Dunnette's multiple comparison *post hoc* tests) analyses were done on prism (GraphPad Software, 2365 Northside Dr., Suite 560 San Diego, CA 92108 USA).

## Conflict of interest

The authors declare no conflict of interest.

## Author contributions

OU, AVZ, DBP, MNP, and IT conceived the study. OU and AVZ, designed and conducted experiments on gene expression, cell functioning, migration, signaling, and metabolism. DEA and PVB carried out RNA‐sequence analysis. LH, DEA and DJP assisted with metabolic experiments. PJ carried out the transwell migration assay and analysis. YW and PH carried out ELISA and Western blotting experiments. AVZ and OU wrote the initial draft of the manuscript. DBP, MNP, and IT provided funding. All authors contributed to the interpretation of the data and editing the final version of the manuscript.

## Supporting information


**Fig. S1**. *SCO2* regulates glucose metabolism. A‐D, Glucose uptake (A and C) and extracellular lactate levels (B and D) were monitored using BioProfiler (QIAGEN Redwood City, 1001 Marshall Street, Redwood City, CA 94063, United States) analyzer in the indicated cell lines. Data are represented as mean fold change relative to the scrambled shRNA (SCR) control in A549 cells (A‐B) or empty vector (EV) control in HCT116 cells (C‐D) [SCR A549 and WT EV HCT116; set to 1 ± standard deviation (SD)]. ****P < 0.0001, ***P < 0.001 ((A‐B) Unpaired 2‐tailed *t*‐test; *n* = 2 independent experiments with 3 technical replicates each. (C‐D) One‐way ANOVA; Tukey's multiple comparison *post hoc* test; *n* = 4 independent experiments with 1‐3 technical replicates in each). E. Levels of SCO2 in WT and *SCO2* ^−/−^ HCT116 cells infected with an empty vector (EV) or *SCO2* ^−/−^ HCT116 cells where *SCO2* was re‐expressed (+SCO2) was monitored by Western blotting (*N* = 3 independent experiments). α‐tubulin served as a loading control. Quantifications of the Western blots are provided in Fig. S9.
**Fig. S2**. Inhibition of complex III or IV of the electron transport chain results in lipid droplet accumulation in HCT116 cells. A‐C, Confocal microscopy of lipid droplets using Nile Red staining with the fluorescent images composed of stacks of 5 focal planes taken with 0.5 µm step subsequently superimposed with single‐plane DIC images (*N* = 20 cells for each condition). (A) *SCO2* ^−/−^ HCT116 cells were grown for 10 and 20 days in the presence of regular FA^+^ and FA‐deficient FBS (FA‐). B. *SCO2* ^−/−^ and WT HCT116 cells were maintained for 10 days at 19%, 3% or 0.1% O_2_. C. WT HCT116 cells were treated with complex III inhibitors antimycin A (Ant A, 5 μm) or myxothiazol (Myx, 2 μm) for 10 days. All results are presented as median and interquartile range (boxes) (A‐C) along with individual data points (B‐C). Mean value obtained for WT HCT116 cells was set to 1 a.u. ****P < 0.0001 (Unpaired 2‐tailed *t*‐test).
**Fig. S3**. HCT116 cells are devoid of mutations in *IDH1* and *IDH2* genes while the loss of *SCO2* does not affect intracellular pH. A. Sequence analysis of *IDH1* and *IDH2* genes in WT and *SCO2* ^−/−^ HCT116 cells. Complete amino acid sequences of IDH1 and IDH2 proteins and DNA fragments containing the known mutations (R140, R149 and R172) that underlie D‐2‐HG production by IDH is provided. The absence of mutations was confirmed by RNAseq analysis (*n* = 2 independent experiments). B. The effects of *SCO2* on intracellular pH were assessed via confocal FLIM of 2′‐7′‐bis(carboxyethyl)‐5(6)‐carboxyfluorescein (BCECF) staining. Images are stacks of three focal planes taken with a 1 µm step. Right panel shows cumulative distributions of BCECF lifetime values in cells localized in the entire field of view (256 × 256 pixels); dotted lines demonstrate lifetime values that correspond to the 50^th^ percentile (median) of the lifetime distribution (*n* = 3 independent experiments).
**Fig. S4**. *SCO2* deletion in HCT116 cells increases expression of SOD2. A. Levels of indicated proteins were monitored by Western blotting. VDAC1 and α‐tubulin served as loading controls. The experiments are representative of 3 independent replicates. Quantifications of the Western blots are provided in Fig. S9.
**Fig. S5**. *SCO2* loss rewires signaling, reduces proliferation and bolsters migration A‐C, Levels and phosphorylation status of indicated proteins were monitored by Western blotting after lentiviral knockdown of SCO2 (shSCO2) in A549 (A), HeLa (B) and HT29 (C) cells. In parallel, A549 (A), HeLa (B) and HT29 (C) cells were infected with scrambled shRNA (SCR). Shown are the representative Western blots from 3 (A549 and HeLa) or 2 (HT29) independent experiments. α‐tubulin was used as a loading control. D‐F. Numbers of viable SCR (red) and shSCO2 (blue) infected A549 (D), HeLa (E) and HT29 (F) cells grown for indicated times were determined by trypan blue exclusion using automated cell counter. Results are presented as the mean value ± SD whereby the number of SCR cells at day 1 was set to 1 (*n* = 2 independent experiments with 3 technical replicates each for A549, *n* = 4 independent experiments with 3 technical replicates each for HeLa, *n* = 3 independent experiments with 3 technical replicates each for HT29). G. Levels and phosphorylation status of indicated proteins in WT empty vector (EV), *SCO2* ^−/−^ EV and *SCO2* ^−/−^ cells in which SCO2 was re‐expressed (+SCO2) HCT116 cells were monitored by Western blotting. α‐tubulin served as a loading control. Shown are representative Western blots from 3 independent experiments. H. Numbers of WT and *SCO2* ^−/−^ HCT116 cells infected with empty vector (EV) and *SCO2* ^−/−^ HCT116 cells re‐expressing *SCO2* (+SCO2) grown for 3 days were assessed using trypan blue exclusion using automated cell counter. Results are presented as the mean value ± SD relative to WT EV HCT116 cell number, which was set to 1 (*n* = 2 independent experiments with 2‐3 technical replicates each). I. Transwell migration assay of WT EV, *SCO2* ^−/−^EV and *SCO2* ^−/−^(+SCO2) HCT116 cells. Presented are the mean summarized cell counts of four fields from a single transwell migration chamber ± SD (****P < 0.0001; ***P < 0.001 One‐way ANOVA; Tukey's multiple comparison *post hoc* test; *n* = 2 independent experiments with 2 technical replicates in each). J. Levels and phosphorylation status of indicated proteins were monitored by Western blotting in WT EV, *SCO2* ^−/−^ EV and *SCO2* ^−/−^ (+SCO2) HCT116 cells. α‐tubulin served as a loading control. Shown are representative Western blots from 3 independent experiments. K. Levels and phosphorylation status of indicated proteins were monitored by Western blotting in WT and *SCO2* ^−/−^ HCT116 cells. α‐tubulin served as a loading control. Shown are representative Western blots from 4 independent experiments. Quantifications of the Western blots are provided in Fig. S9.
**Fig. S6**. Reduced complex IV activity increases sensitivity cancer cells to AKT inhibitor. A‐B. A549 (A) and HT29 (B) cells were infected with scrambled shRNA (SCR) or SCO2 shRNA (shSCO2). The cells were treated with indicated concentrations of pan‐AKT inhibitor MK2206 or a vehicle (DMSO) for 3 days. The number of viable cells were determined using trypan blue exclusion and automated cell counter. Data are presented as the mean fraction of cells relative to the corresponding DMSO‐treated controls, which were set to 1. Experiments were carried out in at least 2 independent replicates (with 2 technical replicates each). Bars represent SD values. Data are represented as mean fold change ± SD relative to vehicle (DMSO) treated cells. *P < 0.05, **P < 0.01, ***P < 0.001, ****P < 0.0001 (Unpaired 2‐tailed *t*‐tests were carried out at each drug concentration tested).
**Fig. S7**. Inhibition of AKT dampens the compensatory increase in glycolysis caused by the deletion of *SCO2* in HCT116 cells. A‐B, Intracellular levels of DHAP (A) and 3‐PG (B) in WT or *SCO2* ^−/−^ HCT116 cells. Cells were seeded and treated with MK2206 (15 µm) for 24 h. Metabolite levels were monitored by GC‐MS. Data are represented as mean fold change ± SD relative to vehicle (DMSO) treated WT HCT116 cells. *P < 0.05, **P < 0.01, ***P < 0.001, ****P < 0.0001 (One‐way ANOVA; Tukey's multiple comparison *post hoc* test; *n* = 2 independent experiments with 3 technical replicates in each).
**Fig. S8**. Depletion of mitochondrial complex IV activity reduces sensitivity of cancer cells to mTOR inhibitors. A549 (A‐B) and HT29 (C‐D) cells were infected with scrambled shRNA (SCR) or SCO2 shRNA (shSCO2). The cells were treated with indicated concentrations of torin 1 (A, B) or rapamycin (C, D) or a vehicle (DMSO) for 3 days. Numbers of viable cells in each condition were determined by trypan blue exclusion using automated cell counter. Data are represented as the mean fraction of cells relative to DMSO‐treated controls ± SD. Experiments were carried out in at least independent duplicate with 2 technical replicates each. Error bars represent SD values. *P < 0.05, **P < 0.01, ***P < 0.001, ****P < 0.0001 (Unpaired 2‐tailed *t*‐tests were carried out at each drug concentration tested).
**Fig. S9**. Densitometry analysis of Western blots. Results represent mean fold changes in densitometry signals relative to the indicated controls. Signals for phosphoproteins and proteins of interest were normalized over corresponding total proteins or α‐tubulin, respectively as indicated. The experiments were repeated at least in independent duplicates; *P < 0.05, **P < 0.01, ***P < 0.001, ****P < 0.0001 (Unpaired 2‐tailed *t*‐test, 1‐way ANOVAs, and 2‐way ANOVAs were done when two, three or more than three variables were investigated, respectively).
**Fig. S10.** Images and scans of Western blot films that are incorporated in the manuscript. The boxes around the indicated protein bands show the approximate area of the x‐ray films and nitrocellulose membrane images included in the figures.

## Data Availability

Raw data are deposited at Mendeley (https://data.mendeley.com/datasets/pyr8t5ng6r/draft?a=754177d8‐d6f3‐4de6‐85b9‐645dd4136d2f).

## References

[1] Vasan K , Werner M , Chandel NS . Mitochondrial metabolism as a target for cancer therapy. Cell Metab. 2020;32:341–52. 32668195 10.1016/j.cmet.2020.06.019 PMC7483781

[2] Hollinshead KER , Parker SJ , Eapen VV , Encarnacion‐Rosado J , Sohn A , Oncu T , et al. Respiratory supercomplexes promote mitochondrial efficiency and growth in severely hypoxic pancreatic cancer. Cell Rep. 2020;33:108231. 33027658 10.1016/j.celrep.2020.108231 PMC7573785

[3] Janeway KA , Kim SY , Lodish M , Nosé V , Rustin P , Gaal J , et al. Defects in succinate dehydrogenase in gastrointestinal stromal tumors lacking KIT and PDGFRA mutations. Proc Natl Acad Sci USA. 2011;108:314–8. 21173220 10.1073/pnas.1009199108 PMC3017134

[4] Astuti D , Latif F , Dallol A , Dahia PL , Douglas F , George E , et al. Gene mutations in the succinate dehydrogenase subunit SDHB cause susceptibility to familial pheochromocytoma and to familial paraganglioma. Am J Hum Genet. 2001;69:49–54. 11404820 10.1086/321282 PMC1226047

[5] Balss J , Meyer J , Mueller W , Korshunov A , Hartmann C , von Deimling A . Analysis of the IDH1 codon 132 mutation in brain tumors. Acta Neuropathol. 2008;116:597–602. 18985363 10.1007/s00401-008-0455-2

[6] Dang L , White DW , Gross S , Bennett BD , Bittinger MA , Driggers EM , et al. Cancer‐associated IDH1 mutations produce 2‐hydroxyglutarate. Nature. 2010;465:966. 20559394 10.1038/nature09132 PMC3766976

[7] Tomlinson IP , Alam NA , Rowan AJ , Barclay E , Jaeger EE , Kelsell D , et al. Germline mutations in FH predispose to dominantly inherited uterine fibroids, skin leiomyomata and papillary renal cell cancer. Nat Genet. 2002;30:406–10. 11865300 10.1038/ng849

[8] Baksh SC , Finley LWS . Metabolic coordination of cell fate by a‐ketoglutarate‐dependent dioxygenases. Trends Cell Biol. 2021;31:24–36. 33092942 10.1016/j.tcb.2020.09.010 PMC7748998

[9] Leary SC , Cobine PA , Kaufman BA , Guercin GH , Mattman A , Palaty J , et al. The human cytochrome c oxidase assembly factors SCO1 and SCO2 have regulatory roles in the maintenance of cellular copper homeostasis. Cell Metab. 2007;5:9–20. 17189203 10.1016/j.cmet.2006.12.001

[10] Chinnery PF , Keogh MJ . Clinical mitochondrial medicine. Cambridge University Press; 2018.

[11] Ghezzi D , Zeviani M . Human diseases associated with defects in assembly of OXPHOS complexes. Essays Biochem. 2018;62:271–86. 30030362 10.1042/EBC20170099 PMC6056716

[12] Matoba S , Kang JG , Patino WD , Wragg A , Boehm M , Gavrilova O , et al. p53 regulates mitochondrial respiration. Science. 2006;312:1650–3. 16728594 10.1126/science.1126863

[13] Sung HJ , Ma W , Wang PY , Hynes J , O'Riordan TC , Combs CA , et al. Mitochondrial respiration protects against oxygen‐associated DNA damage. Nat Commun. 2010;1:5. 20975668 10.1038/ncomms1003 PMC3393093

[14] Zhdanov AV , Andreev DE , Baranov PV , Papkovsky DB . Low energy costs of F1Fo ATP synthase reversal in colon carcinoma cells deficient in mitochondrial complex IV. Free Radic Biol Med. 2017;106:184–95. 28189850 10.1016/j.freeradbiomed.2017.02.025

[15] Matsumoto T , Wang PY , Ma W , Sung HJ , Matoba S , Hwang PM . Polo‐like kinases mediate cell survival in mitochondrial dysfunction. Proc Natl Acad Sci USA. 2009;106:14542–6. 19706541 10.1073/pnas.0904229106 PMC2732832

[16] Wang L , Xiong H , Wu F , Zhang Y , Wang J , Zhao L , et al. Hexokinase 2‐mediated Warburg effect is required for PTEN‐ and p53‐deficiency‐driven prostate cancer growth. Cell Rep. 2014;8:1461–74. 25176644 10.1016/j.celrep.2014.07.053 PMC4360961

[17] Kondoh H , Lleonart ME , Gil J , Wang J , Degan P , Peters G , et al. Glycolytic enzymes can modulate cellular life span. Cancer Res. 2005;65:177–85. 15665293

[18] Bensaad K , Tsuruta A , Selak MA , Vidal MN , Nakano K , Bartrons R , et al. TIGAR, a p53‐inducible regulator of glycolysis and apoptosis. Cell. 2006;126:107–20. 16839880 10.1016/j.cell.2006.05.036

[19] Wanka C , Brucker DP , Bähr O , Ronellenfitsch M , Weller M , Steinbach JP , et al. Synthesis of cytochrome C oxidase 2: a p53‐dependent metabolic regulator that promotes respiratory function and protects glioma and colon cancer cells from hypoxia‐induced cell death. Oncogene. 2012;31:3764–76. 22120717 10.1038/onc.2011.530

[20] Gyorffy B , Lánczky A , Szállási Z . Implementing an online tool for genome‐wide validation of survival‐associated biomarkers in ovarian‐cancer using microarray data from 1287 patients. Endocr Relat Cancer. 2012;19:197–208. 22277193 10.1530/ERC-11-0329

[21] Won KY , Lim SJ , Kim GY , Kim YW , Han SA , Song JY , et al. Regulatory role of p53 in cancer metabolism via SCO2 and TIGAR in human breast cancer. Hum Pathol. 2012;43:221–8. 21820150 10.1016/j.humpath.2011.04.021

[22] Alldredge J , Randall L , De Robles G , Agrawal A , Mercola D , Liu M , et al. Transcriptome analysis of ovarian and uterine clear cell malignancies. Front Oncol. 2020;10:598579. 33415077 10.3389/fonc.2020.598579 PMC7784081

[23] Nath A , Chan C . Genetic alterations in fatty acid transport and metabolism genes are associated with metastatic progression and poor prognosis of human cancers. Sci Rep. 2016;6:18669. 26725848 10.1038/srep18669 PMC4698658

[24] Hulea L , Gravel SP , Morita M , Cargnello M , Uchenunu O , Im YK , et al. Translational and HIF‐1α‐dependent metabolic reprogramming underpin metabolic plasticity and responses to kinase inhibitors and biguanides. Cell Metab. 2018;28(6):817–32.e8. 30244971 10.1016/j.cmet.2018.09.001 PMC7252493

[25] Janzer A , German NJ , Gonzalez‐Herrera KN , Asara JM , Haigis MC , Struhl K . Metformin and phenformin deplete tricarboxylic acid cycle and glycolytic intermediates during cell transformation and NTPs in cancer stem cells. Proc Natl Acad Sci USA. 2014;111:10574–9. 25002509 10.1073/pnas.1409844111 PMC4115496

[26] Brisson D , Vohl MC , St‐Pierre J , Hudson TJ , Gaudet D . Glycerol: a neglected variable in metabolic processes? BioEssays. 2001;23:534–42. 11385633 10.1002/bies.1073

[27] Williamson DH , Lund P , Krebs HA . The redox state of free nicotinamide‐adenine dinucleotide in the cytoplasm and mitochondria of rat liver. Biochem J. 1967;103:514–27. 4291787 10.1042/bj1030514 PMC1270436

[28] Pettit FH , Pelley JW , Reed LJ . Regulation of pyruvate dehydrogenase kinase and phosphatase by acetyl‐CoA/CoA and NADH/NAD ratios. Biochem Biophys Res Commun. 1975;65:575–82. 167775 10.1016/s0006-291x(75)80185-9

[29] Birsoy K , Wang T , Chen WW , Freinkman E , Abu‐Remaileh M , Sabatini DM . An essential role of the mitochondrial electron transport chain in cell proliferation is to enable aspartate synthesis. Cell. 2015;162:540–51. 26232224 10.1016/j.cell.2015.07.016 PMC4522279

[30] Sullivan LB , Gui DY , Hosios AM , Bush LN , Freinkman E , Vander Heiden MG . Supporting aspartate biosynthesis is an essential function of respiration in proliferating cells. Cell. 2015;162:552–63. 26232225 10.1016/j.cell.2015.07.017 PMC4522278

[31] Chen WW , Freinkman E , Wang T , Birsoy K , Sabatini DM . Absolute quantification of matrix metabolites reveals the dynamics of mitochondrial metabolism. Cell. 2016;166:1324–37.e11. 27565352 10.1016/j.cell.2016.07.040 PMC5030821

[32] Patel MS , Nemeria NS , Furey W , Jordan F . The pyruvate dehydrogenase complexes: structure‐based function and regulation. J Biol Chem. 2014;289:16615–23. 24798336 10.1074/jbc.R114.563148 PMC4059105

[33] Denton RM , Randle PJ , Bridges BJ , Cooper RH , Kerbey AL , Pask HT , et al. Regulation of mammalian pyruvate dehydrogenase. Mol Cell Biochem. 1975;9:27–53. 171557 10.1007/BF01731731

[34] Rardin MJ , Wiley SE , Naviaux RK , Murphy AN , Dixon JE . Monitoring phosphorylation of the pyruvate dehydrogenase complex. Anal Biochem. 2009;389:157–64. 19341700 10.1016/j.ab.2009.03.040 PMC2713743

[35] Korotchkina LG , Patel MS . Site specificity of four pyruvate dehydrogenase kinase isoenzymes toward the three phosphorylation sites of human pyruvate dehydrogenase. J Biol Chem. 2001;276:37223–9. 11486000 10.1074/jbc.M103069200

[36] Kolobova E , Tuganova A , Boulatnikov I , Popov KM . Regulation of pyruvate dehydrogenase activity through phosphorylation at multiple sites. Biochem J. 2001;358:69–77. 11485553 10.1042/0264-6021:3580069 PMC1222033

[37] Donaldson WE . Regulation of fatty acid synthesis. Fed Proc. 1979;38:2617–21. 40828

[38] Hill S , Deepa SS , Sataranatarajan K , Premkumar P , Pulliam D , Liu Y , et al. Sco2 deficient mice develop increased adiposity and insulin resistance. Mol Cell Endocrinol. 2017;455:103–14. 28428045 10.1016/j.mce.2017.03.019 PMC5592144

[39] Schönenberger MJ , Kovacs WJ . Hypoxia signaling pathways: modulators of oxygen‐related organelles. Front Cell Dev Biol. 2015;3:42. 26258123 10.3389/fcell.2015.00042 PMC4508581

[40] Tretter L , Patocs A , Chinopoulos C . Succinate, an intermediate in metabolism, signal transduction, ROS, hypoxia, and tumorigenesis. Biochim Biophys Acta. 2016;1857:1086–101. 26971832 10.1016/j.bbabio.2016.03.012

[41] Selak MA , Armour SM , MacKenzie ED , Boulahbel H , Watson DG , Mansfield KD , et al. Succinate links TCA cycle dysfunction to oncogenesis by inhibiting HIF‐alpha prolyl hydroxylase. Cancer Cell. 2005;7:77–85. 15652751 10.1016/j.ccr.2004.11.022

[42] Hobert JA , Mester JL , Moline J , Eng C . Elevated plasma succinate in PTEN, SDHB, and SDHD mutation‐positive individuals. Genet Med. 2012;14:616–9. 22261759 10.1038/gim.2011.63 PMC4019996

[43] Letouzé E , Martinelli C , Loriot C , Burnichon N , Abermil N , Ottolenghi C , et al. SDH mutations establish a hypermethylator phenotype in paraganglioma. Cancer Cell. 2013;23:739–52. 23707781 10.1016/j.ccr.2013.04.018

[44] Mamer O , Gravel SP , Choinière L , Chénard V , St‐Pierre J , Avizonis D . The complete targeted profile of the organic acid intermediates of the citric acid cycle using a single stable isotope dilution analysis, sodium borodeuteride reduction and selected ion monitoring GC/MS. Metabolomics. 2013;9:1019–30. 24348278 10.1007/s11306-013-0521-1 PMC3855487

[45] Ward PS , Patel J , Wise DR , Abdel‐Wahab O , Bennett BD , Coller HA , et al. The common feature of leukemia‐associated IDH1 and IDH2 mutations is a neomorphic enzyme activity converting α‐ketoglutarate to 2‐hydroxyglutarate. Cancer Cell. 2010;17:225–34. 20171147 10.1016/j.ccr.2010.01.020 PMC2849316

[46] Intlekofer AM , Wang B , Liu H , Shah H , Carmona‐Fontaine C , Rustenburg AS , et al. L‐2‐Hydroxyglutarate production arises from noncanonical enzyme function at acidic pH. Nat Chem Biol. 2017;13:494–500. 28263965 10.1038/nchembio.2307 PMC5516644

[47] Intlekofer AM , Dematteo RG , Venneti S , Finley LWS , Lu C , Judkins AR , et al. Hypoxia induces production of L‐2‐hydroxyglutarate. Cell Metab. 2015;22:304–11. 26212717 10.1016/j.cmet.2015.06.023 PMC4527873

[48] Ye D , Guan K‐L , Xiong Y . Metabolism, activity, and targeting of D‐and L‐2‐hydroxyglutarates. Trends Cancer. 2018;4:151–65. 29458964 10.1016/j.trecan.2017.12.005 PMC5884165

[49] Vander Heiden MG , DeBerardinis RJ . Understanding the intersections between metabolism and cancer biology. Cell. 2017;168:657–69. 28187287 10.1016/j.cell.2016.12.039 PMC5329766

[50] Gui DY , Sullivan LB , Luengo A , Hosios AM , Bush LN , Gitego N , et al. Environment dictates dependence on mitochondrial complex I for NAD+ and aspartate production and determines cancer cell sensitivity to metformin. Cell Metab. 2016;24:716–27. 27746050 10.1016/j.cmet.2016.09.006 PMC5102768

[51] Lu SC . Regulation of glutathione synthesis. Mol Aspects Med. 2009;30:42–59. 18601945 10.1016/j.mam.2008.05.005 PMC2704241

[52] Requejo R , Hurd TR , Costa NJ , Murphy MP . Cysteine residues exposed on protein surfaces are the dominant intramitochondrial thiol and may protect against oxidative damage. FEBS J. 2010;277:1465–80. 20148960 10.1111/j.1742-4658.2010.07576.x PMC2847196

[53] Earle WR , Evans VJ , Hawkins NM , Peppers EV , Westfall BB . Effect of glutamine on the growth and metabolism of liver cells *in vitro* . J Natl Cancer Inst. 1956;17:131–8. 13357933

[54] Zhao Y , Zhao X , Chen V , Feng Y , Wang L , Croniger C , et al. Colorectal cancers utilize glutamine as an anaplerotic substrate of the TCA cycle *in vivo* . Sci Rep. 2019;9:19180. 31844152 10.1038/s41598-019-55718-2 PMC6915720

[55] Fendt SM , Bell EL , Keibler MA , Olenchock BA , Mayers JR , Wasylenko TM , et al. Reductive glutamine metabolism is a function of the α‐ketoglutarate to citrate ratio in cells. Nat Commun. 2013;4:2236. 23900562 10.1038/ncomms3236 PMC3934748

[56] Liu GY , Sabatini DM . mTOR at the nexus of nutrition, growth, ageing and disease. Nat Rev Mol Cell Biol. 2020;21:183–203. 31937935 10.1038/s41580-019-0199-y PMC7102936

[57] Gwinn DM , Shackelford DB , Egan DF , Mihaylova MM , Mery A , Vasquez DS , et al. AMPK phosphorylation of raptor mediates a metabolic checkpoint. Mol Cell. 2008;30:214–26. 18439900 10.1016/j.molcel.2008.03.003 PMC2674027

[58] Inoki K , Zhu T , Guan KL . TSC2 mediates cellular energy response to control cell growth and survival. Cell. 2003;115:577–90. 14651849 10.1016/s0092-8674(03)00929-2

[59] Dowling RJ , Topisirovic I , Alain T , Bidinosti M , Fonseca BD , Petroulakis E , et al. mTORC1‐mediated cell proliferation, but not cell growth, controlled by the 4E‐BPs. Science. 2010;328:1172–6. 20508131 10.1126/science.1187532 PMC2893390

[60] Denisenko TV , Gorbunova AS , Zhivotovsky B . Mitochondrial involvement in migration, invasion and metastasis. Front Cell Dev Biol. 2019;7:335. 31921862 10.3389/fcell.2019.00355 PMC6932960

[61] Guerra F , Guaragnella N , Arbini AA , Bucci C , Giannattasio S , Moro L . Mitochondrial dysfunction: a novel potential driver of epithelial‐to‐mesenchymal transition in cancer. Front Oncol. 2017;7:295. 29250487 10.3389/fonc.2017.00295 PMC5716985

[62] Sánchez‐Tilló E , Lázaro A , Torrent R , Cuatrecasas M , Vaquero EC , Castells A , et al. ZEB1 represses E‐cadherin and induces an EMT by recruiting the SWI/SNF chromatin‐remodeling protein BRG1. Oncogene. 2010;29:3490–500. 20418909 10.1038/onc.2010.102

[63] Harvey RF , Pöyry TAA , Stoneley M , Willis AE . Signaling from mTOR to eIF2α mediates cell migration in response to the chemotherapeutic doxorubicin. Sci Signal. 2019;12:eaaw6763. 31848319 10.1126/scisignal.aaw6763

[64] Gandin V , Masvidal L , Cargnello M , Gyenis L , McLaughlan S , Cai Y , et al. mTORC1 and CK2 coordinate ternary and eIF4F complex assembly. Nat Commun. 2016;7:11127. 27040916 10.1038/ncomms11127 PMC4822005

[65] Laplante M , Sabatini DM . An emerging role of mTOR in lipid biosynthesis. Curr Biol. 2009;19:R1046–52. 19948145 10.1016/j.cub.2009.09.058 PMC3390254

[66] Wang X , Proud CG . The mTOR pathway in the control of protein synthesis. Physiology (Bethesda). 2006;21:362–9. 16990457 10.1152/physiol.00024.2006

[67] Faubert B , Solmonson A , DeBerardinis RJ . Metabolic reprogramming and cancer progression. Science (New York, NY). 2020;368:eaaw5473. 10.1126/science.aaw5473 PMC7227780 32273439

[68] DeBerardinis RJ , Lum JJ , Hatzivassiliou G , Thompson CB . The biology of cancer: metabolic reprogramming fuels cell growth and proliferation. Cell Metab. 2008;7:11–20. 18177721 10.1016/j.cmet.2007.10.002

[69] Fan J , Hitosugi T , Chung TW , Xie J , Ge Q , Gu TL , et al. Tyrosine phosphorylation of lactate dehydrogenase A is important for NADH/NAD(+) redox homeostasis in cancer cells. Mol Cell Biol. 2011;31:4938–50. 21969607 10.1128/MCB.06120-11 PMC3233034

[70] Chowdhury SK , Gemin A , Singh G . High activity of mitochondrial glycerophosphate dehydrogenase and glycerophosphate‐dependent ROS production in prostate cancer cell lines. Biochem Biophys Res Commun. 2005;333:1139–45. 15967408 10.1016/j.bbrc.2005.06.017

[71] Elstrom RL , Bauer DE , Buzzai M , Karnauskas R , Harris MH , Plas DR , et al. Akt stimulates aerobic glycolysis in cancer cells. Cancer Res. 2004;64:3892–9. 15172999 10.1158/0008-5472.CAN-03-2904

[72] Kazyken D , Magnuson B , Bodur C , Acosta‐Jaquez HA , Zhang D , Tong X , et al. AMPK directly activates mTORC2 to promote cell survival during acute energetic stress. Sci Signal. 2019;12:eaav3249. 31186373 10.1126/scisignal.aav3249 PMC6935248

[73] Rabinovitch RC , Samborska B , Faubert B , Ma EH , Gravel SP , Andrzejewski S , et al. AMPK maintains cellular metabolic homeostasis through regulation of mitochondrial reactive oxygen species. Cell Rep. 2017;21:1–9. 28978464 10.1016/j.celrep.2017.09.026

[74] Sarbassov DD , Guertin DA , Ali SM , Sabatini DM . Phosphorylation and regulation of Akt/PKB by the rictor‐mTOR complex. Science. 2005;307:1098–101. 15718470 10.1126/science.1106148

[75] Masui K , Tanaka K , Akhavan D , Babic I , Gini B , Matsutani T , et al. mTOR complex 2 controls glycolytic metabolism in glioblastoma through FoxO acetylation and upregulation of c‐Myc. Cell Metab. 2013;18:726–39. 24140020 10.1016/j.cmet.2013.09.013 PMC3840163

[76] Woods A , Dickerson K , Heath R , Hong SP , Momcilovic M , Johnstone SR , et al. Ca2+/calmodulin‐dependent protein kinase kinase‐beta acts upstream of AMP‐activated protein kinase in mammalian cells. Cell Metab. 2005;2:21–33. 16054096 10.1016/j.cmet.2005.06.005

[77] Jiang J , Wang K , Chen Y , Chen H , Nice EC , Huang C . Redox regulation in tumor cell epithelial–mesenchymal transition: molecular basis and therapeutic strategy. Signal Transduct Target Ther. 2017;2:17036. 29263924 10.1038/sigtrans.2017.36 PMC5661624

[78] Colvin H , Nishida N , Konno M , Haraguchi N , Takahashi H , Nishimura J , et al. Oncometabolite D‐2‐hydroxyglurate directly induces epithelial‐mesenchymal transition and is associated with distant metastasis in colorectal cancer. Sci Rep. 2016;6:36289. 27824159 10.1038/srep36289 PMC5099755

[79] Craene BD , Berx G . Regulatory networks defining EMT during cancer initiation and progression. Nat Rev Cancer. 2013;13:97–110. 23344542 10.1038/nrc3447

[80] Yang J , Weinberg RA . Epithelial‐mesenchymal transition: at the crossroads of development and tumor metastasis. Dev Cell. 2008;14:818–29. 18539112 10.1016/j.devcel.2008.05.009

[81] Gravel S‐P , Avizonis D , St‐Pierre J . Metabolomics analyses of cancer cells in controlled microenvironments. Methods Mol Biol (Clifton, NJ). 2016;1458:273–90. 10.1007/978-1-4939-3801-8_20 27581029

[82] Andreev DE , O'Connor PB , Fahey C , Kenny EM , Terenin IM , Dmitriev SE , et al. Translation of 5′ leaders is pervasive in genes resistant to eIF2 repression. Elife. 2015;4:e03971. 25621764 10.7554/eLife.03971 PMC4383229

[83] Zhdanov AV , Okkelman IA , Golubeva AV , Doerr B , Hyland NP , Melgar S , et al. Quantitative analysis of mucosal oxygenation using ex vivo imaging of healthy and inflamed mammalian colon tissue. Cell Mol Life Sci. 2017;74:141–51. 27510419 10.1007/s00018-016-2323-x PMC11107550

