# Supplementary material for: Mitochondrial complex IV defects induce metabolic and signaling perturbations that expose potential vulnerabilities in HCT116 cells
Source: FEBS Open Bio. 2022 Apr 1;12(5):959–82. doi: 10.1002/2211-5463.13398 (PMC9063438; doi:10.1002/2211-5463.13398)
Supplement: Supplementary file 1 — Fig. S1. SCO2 regulates glucose metabolism. A‐D, Glucose uptake (A and C) and extracellular lactate levels (B and D) were monitored using BioProfiler (QIAGEN Redwood City, 1001 Marshall Street, Redwood City, CA 94063, United States) analyzer in the indicated cell lines. Data are represented as mean fold change relative to the scrambled shRNA (SCR) control in A549 cells (A‐B) or empty vector (EV) control in HCT116 cells (C‐D) [SCR A549 and WT EV HCT116; set to 1 ± standard deviation (SD)]. ****P < 0.0001, ***P < 0.001 ((A‐B) Unpaired 2‐tailed t‐test; n = 2 independent experiments with 3 technical replicates each. (C‐D) One‐way ANOVA; Tukey's multiple comparison post hoc test; n = 4 independent experiments with 1‐3 technical replicates in each). E. Levels of SCO2 in WT and SCO2 −/− HCT116 cells infected with an empty vector (EV) or SCO2 −/− HCT116 cells where SCO2 was re‐expressed (+SCO2) was monitored by Western blotting (N = 3 independent experiments). α‐tubulin served as a loading control. Quantifications of the Western blots are provided in Fig. S9. Fig. S2. Inhibition of complex III or IV of the electron transport chain results in lipid droplet accumulation in HCT116 cells. A‐C, Confocal microscopy of lipid droplets using Nile Red staining with the fluorescent images composed of stacks of 5 focal planes taken with 0.5 µm step subsequently superimposed with single‐plane DIC images (N = 20 cells for each condition). (A) SCO2 −/− HCT116 cells were grown for 10 and 20 days in the presence of regular FA+ and FA‐deficient FBS (FA‐). B. SCO2 −/− and WT HCT116 cells were maintained for 10 days at 19%, 3% or 0.1% O2. C. WT HCT116 cells were treated with complex III inhibitors antimycin A (Ant A, 5 μm) or myxothiazol (Myx, 2 μm) for 10 days. All results are presented as median and interquartile range (boxes) (A‐C) along with individual data points (B‐C). Mean value obtained for WT HCT116 cells was set to 1 a.u. ****P < 0.0001 (Unpaired 2‐tailed t‐test). Fig. S3. HCT116 cel [file FEB4-12-959-s001.pdf]

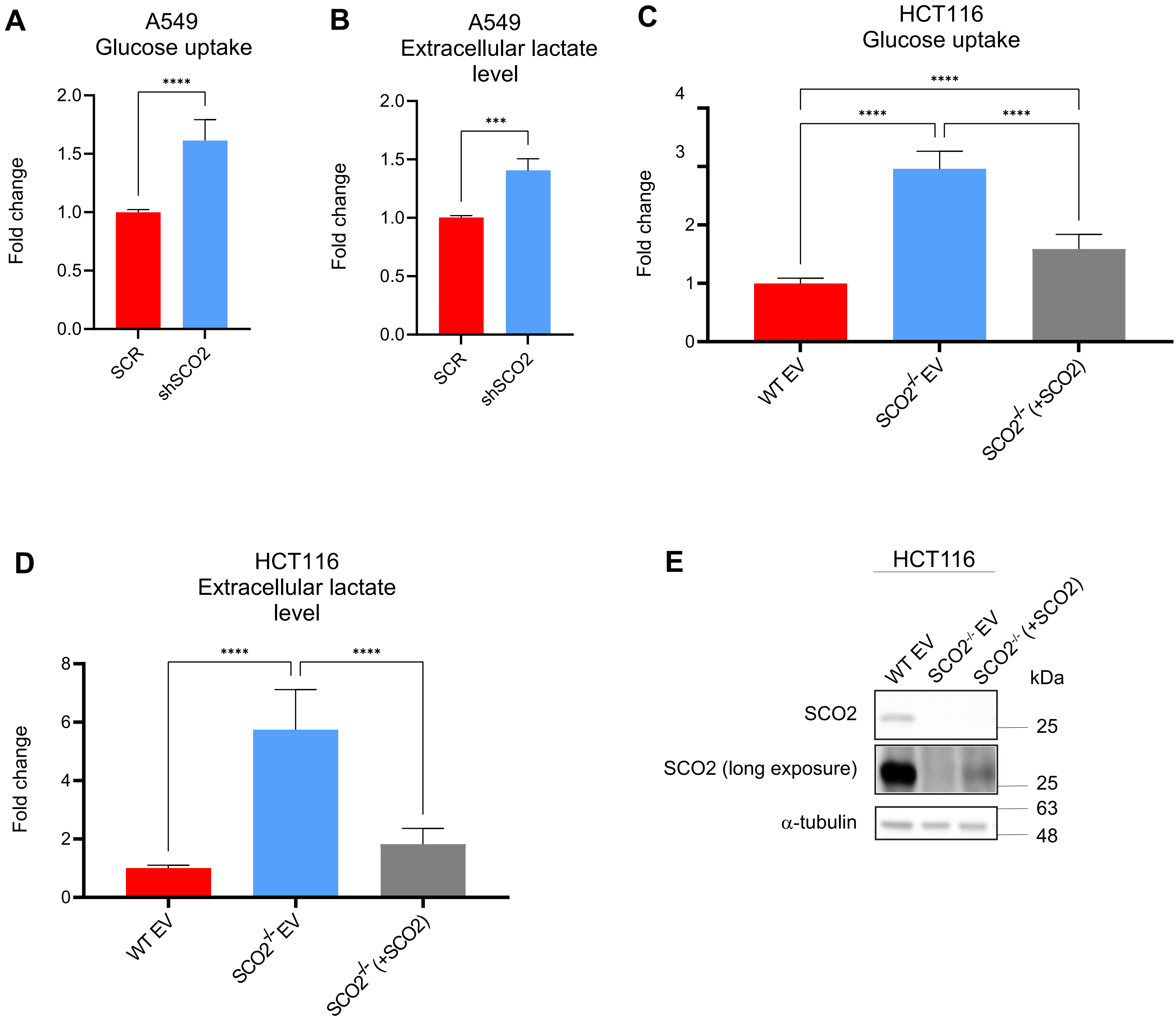

Supplemental Figure 1

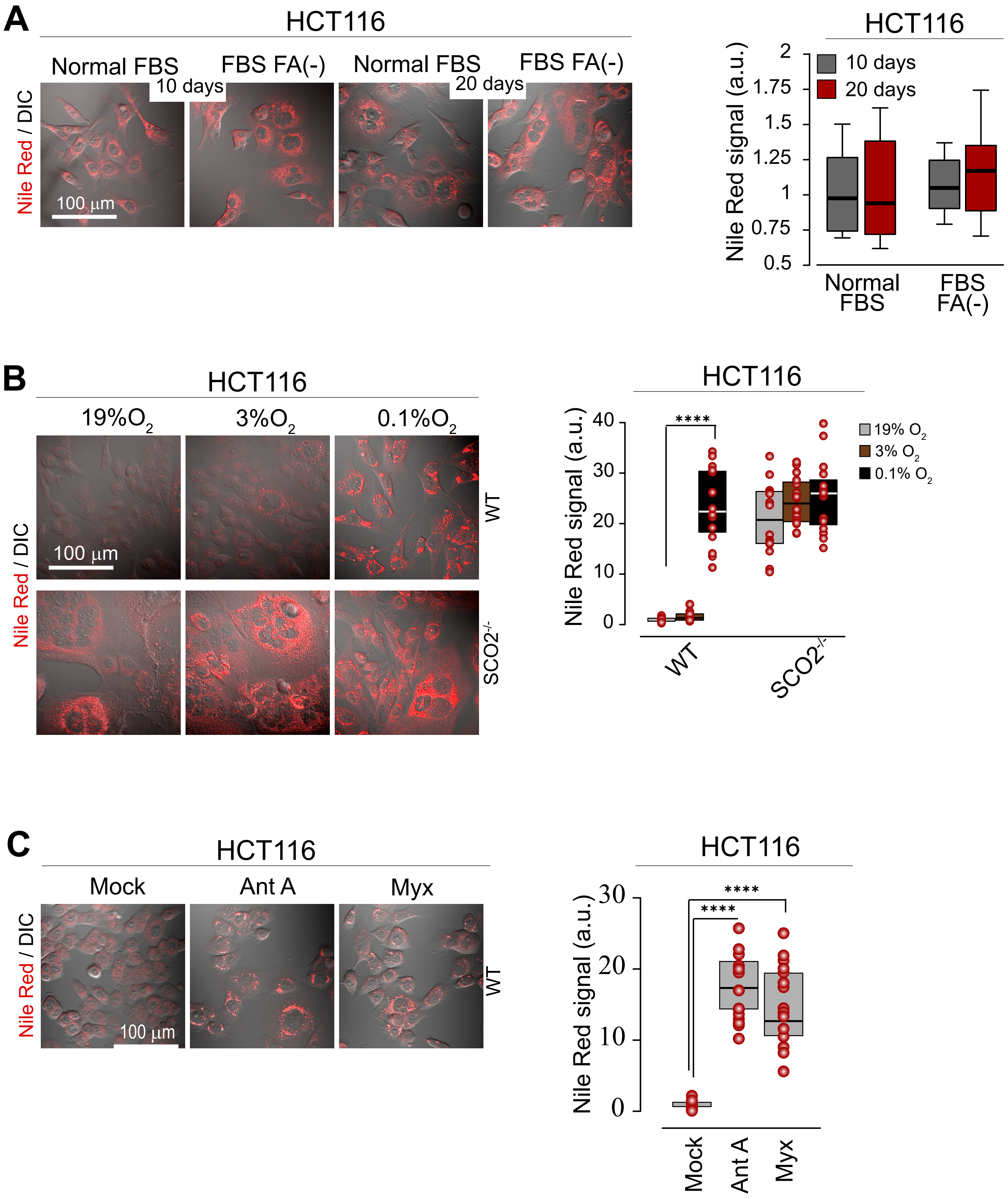

Supplemental Figure 2

A

IDH1

Annotated protein sequence with **R100**, **R109**, **R132** highlighted

MSKKISGGSVVEMQGDEMTRIIWELIKEKLIFPYVELDLHSYDLGIENRDATNDQVTKDAAEAIKKHNVGVKCATITPDEKRVEEFKLKQMWKSPNGTIRNILGGTVFR
EAIICKNIPRLVSGWVKPIIIGRHAYGDQYRATDFVVPGPGKVEITYTPSDGTQKVTYLVHNFEEGGGVAMGMYNQDKSIEDFAHSSFQMALSKGWPLYLSTKNTILKK
YDGRFKDIFQEIYDKQYKSQFEAQKIWYEHRLIDDMVAQAMKSEGGFIWACKNYDGDVQSDSVAQGYGSLGMMTSVLVCPDGKTVEAEAAHGTVTRHYRMYQKG
QETSTNPIASIFAWTRGLAHRAKLDNNKELAFFANALEEVSIETIEAGFMTKDLAACIKGLPNVQRSDYLNTFEFMDKLGENLKIKLAQAKL

R100 R109 R132

WT	…ATA**CGA**AATATTCTGGGTGGCACGGTCTTC**AGA**GAAGCCATTATCTGCAAAAATATCCCCCGGCTTGTGAGTGGATGGGTAAAACCTATCATCATAGGT**CGT**CAT…
SCO2$^{-/-}$	…ATA**CGA**AATATTCTGGGTGGCACGGTCTTC**AGA**GAAGCCATTATCTGCAAAAATATCCCCCGGCTTGTGAGTGGATGGGTAAAACCTATCATCATAGGT**CGT**CAT…

IDH2

Annotated protein sequence with **R140**, **R149**, **R172** highlighted

MAGYLRVVRSLCRASGSRPAWAPAALTAPTSQEQPRRHYADKRIKVAKPVVEMDGDEMTRIIWQFIKEKLILPHVDIQLKYFDLGLPNRDQTDDQVTIDSALATQKYS
VAVKCATITPDEARVEEFKLKKMWKSPNGTIRNILGGTVFREPIICKNIPRLVPGWTKPITIGRHAHGDQYKATDFVADRAGTFKMVFTPKDGSGVKEWEVYNFPAGG
VGMGMYNTDESISGFAHSCFQYAIQKKWPLYMSTKNTILKAYDGRFKDIFQEIFDKHYKTDFDKNKIWYEHRLIDDMVAQVLKSSGGFVWACKNYDGDVQSDILAQ
GFGSLGLMTSVLVCPDGKTIEAEAAHGTVTRHYREHQKGRPTSTNPIASIFAWTRGLEHRGKLDGNQDLIRFAQMLEKVCVETVESGAMTKDLAGCIHGLSNVKLNEH
FLNTTDFLDTIKSNLDRALG

R140 R149 R172

WT	…ACTATC**CGG**AACATCCTGGGGGGGACTGTCTTC**CGG**GAGCCCATCATCTGCAAAAACATCCCACGCCTAGTCCCTGGCTGGACCAAGCCCATCACCATTGGC**AGG**CACGCC…
SCO2$^{-/-}$	…ACTATC**CGG**AACATCCTGGGGGGGACTGTCTTC**CGG**GAGCCCATCATCTGCAAAAACATCCCACGCCTAGTCCCTGGCTGGACCAAGCCCATCACCATTGGC**AGG**CACGCC…

B

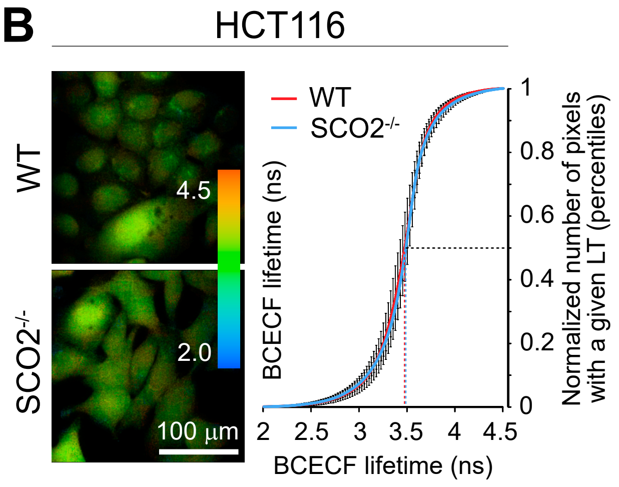

Supplemental Figure 3

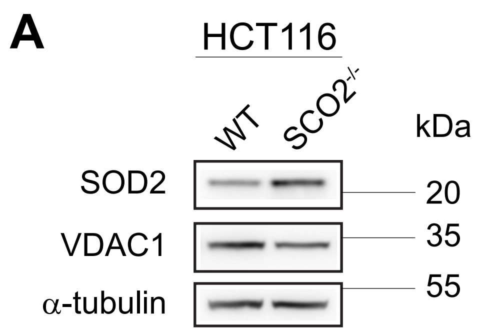

Supplemental Figure 4

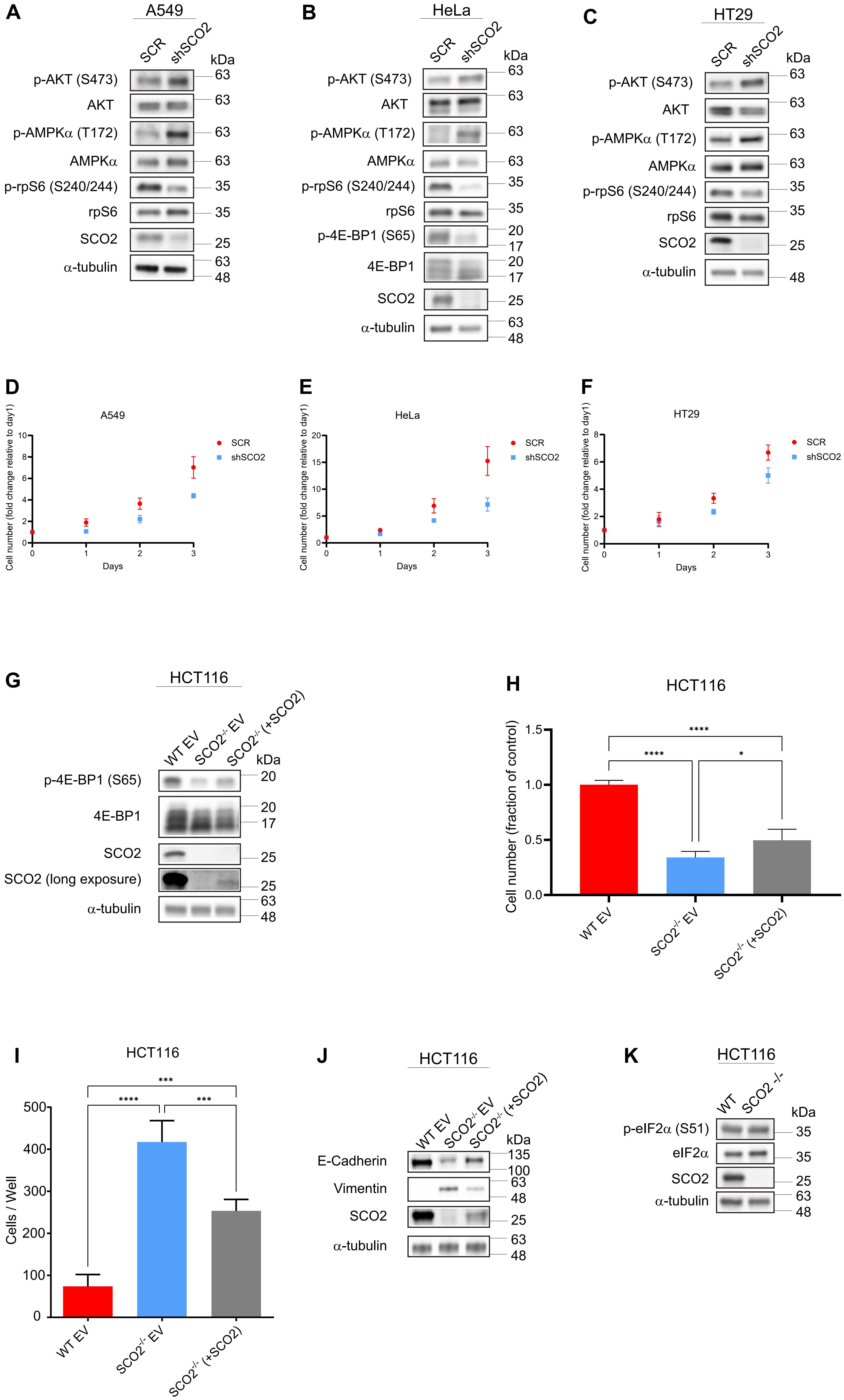

Supplemental Figure 5

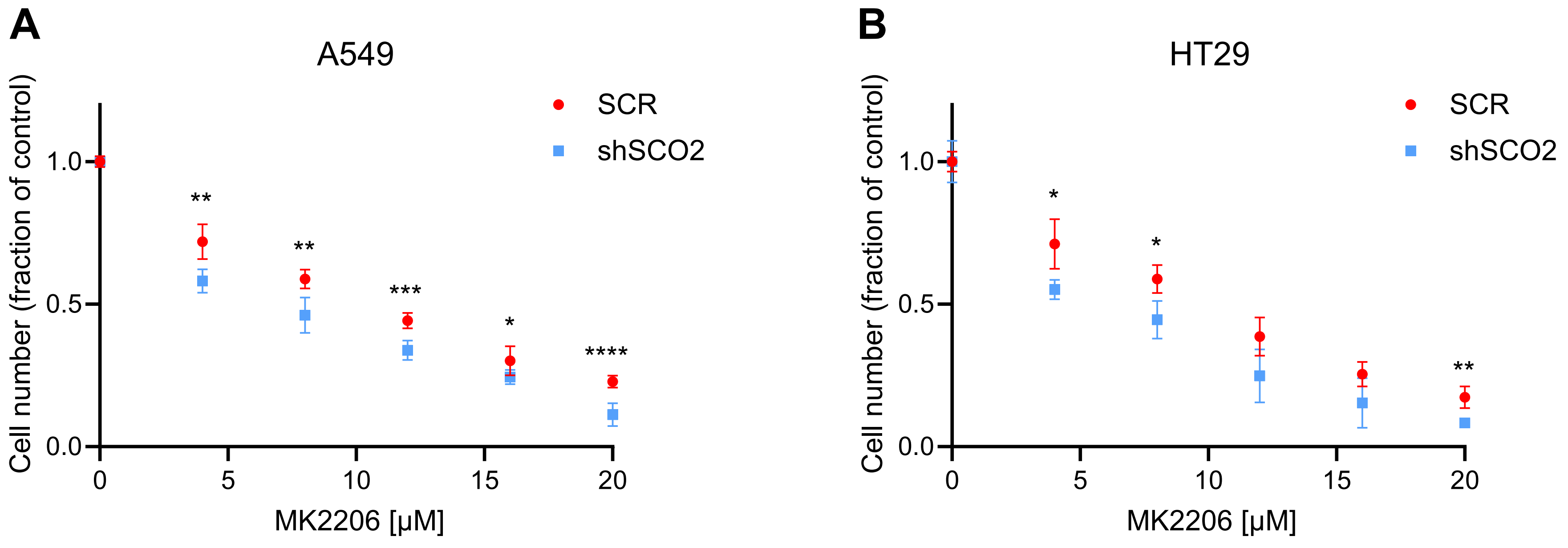

Supplemental Figure 6

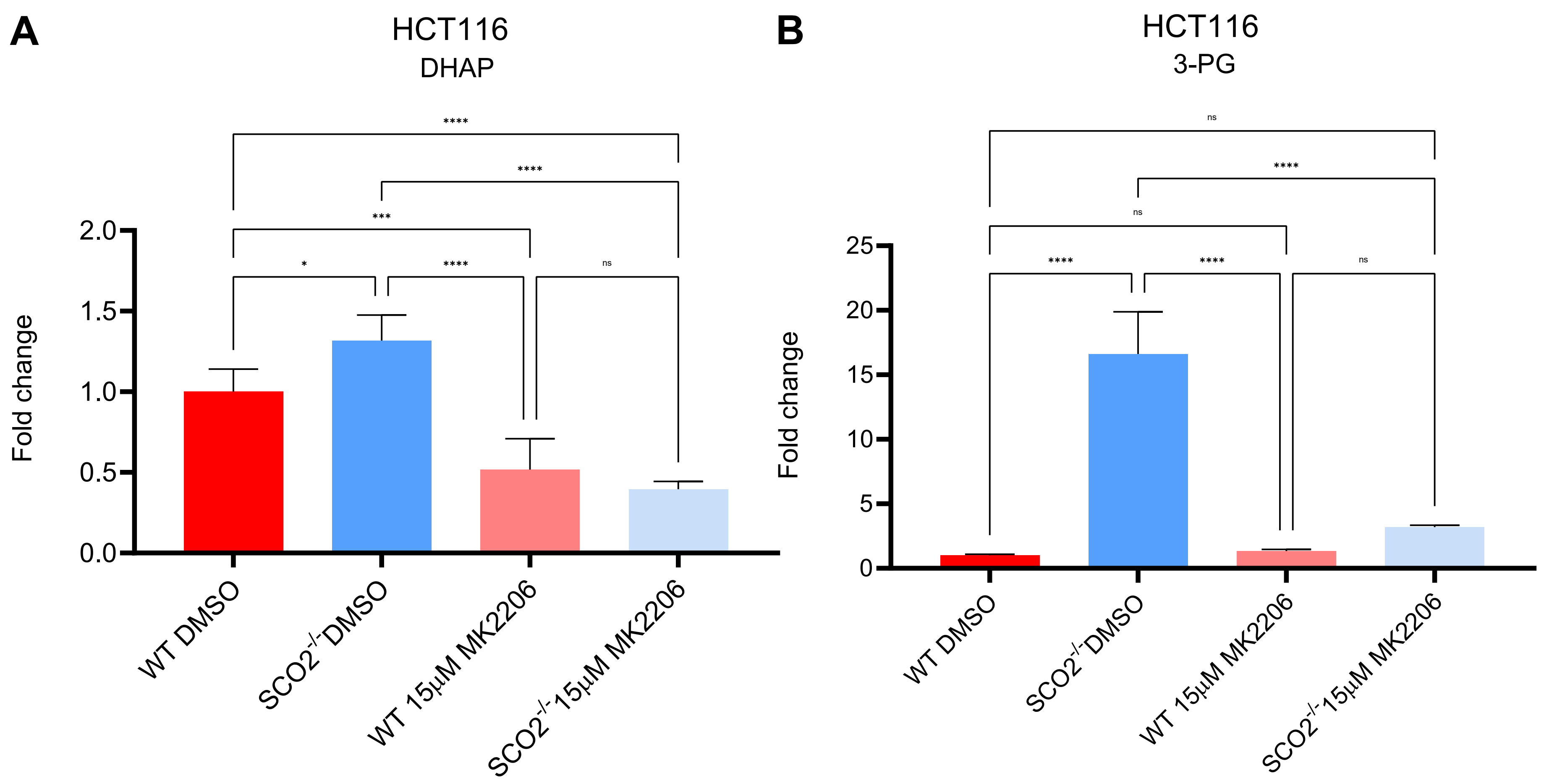

Supplemental Figure 7

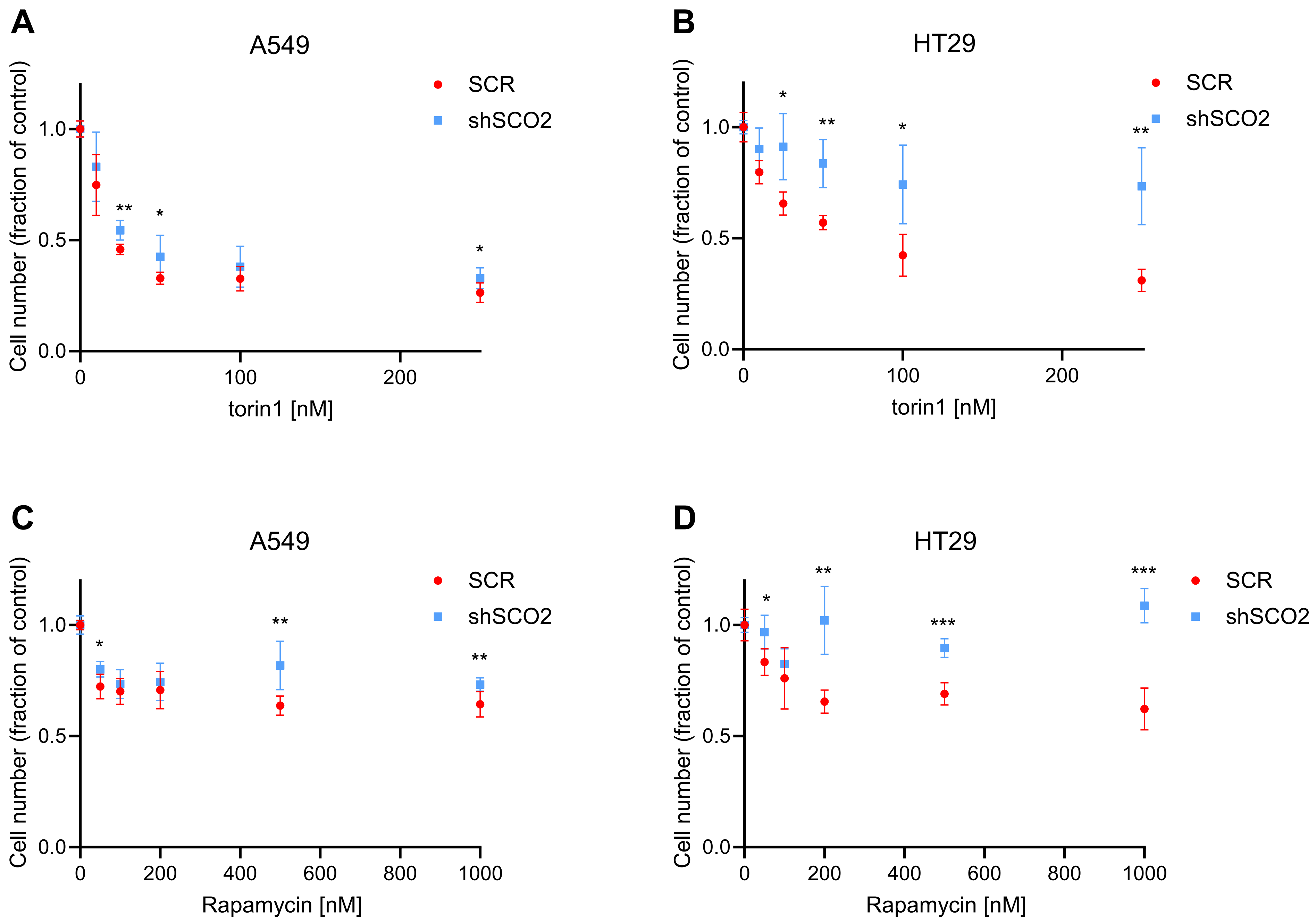

Supplemental Figure 8

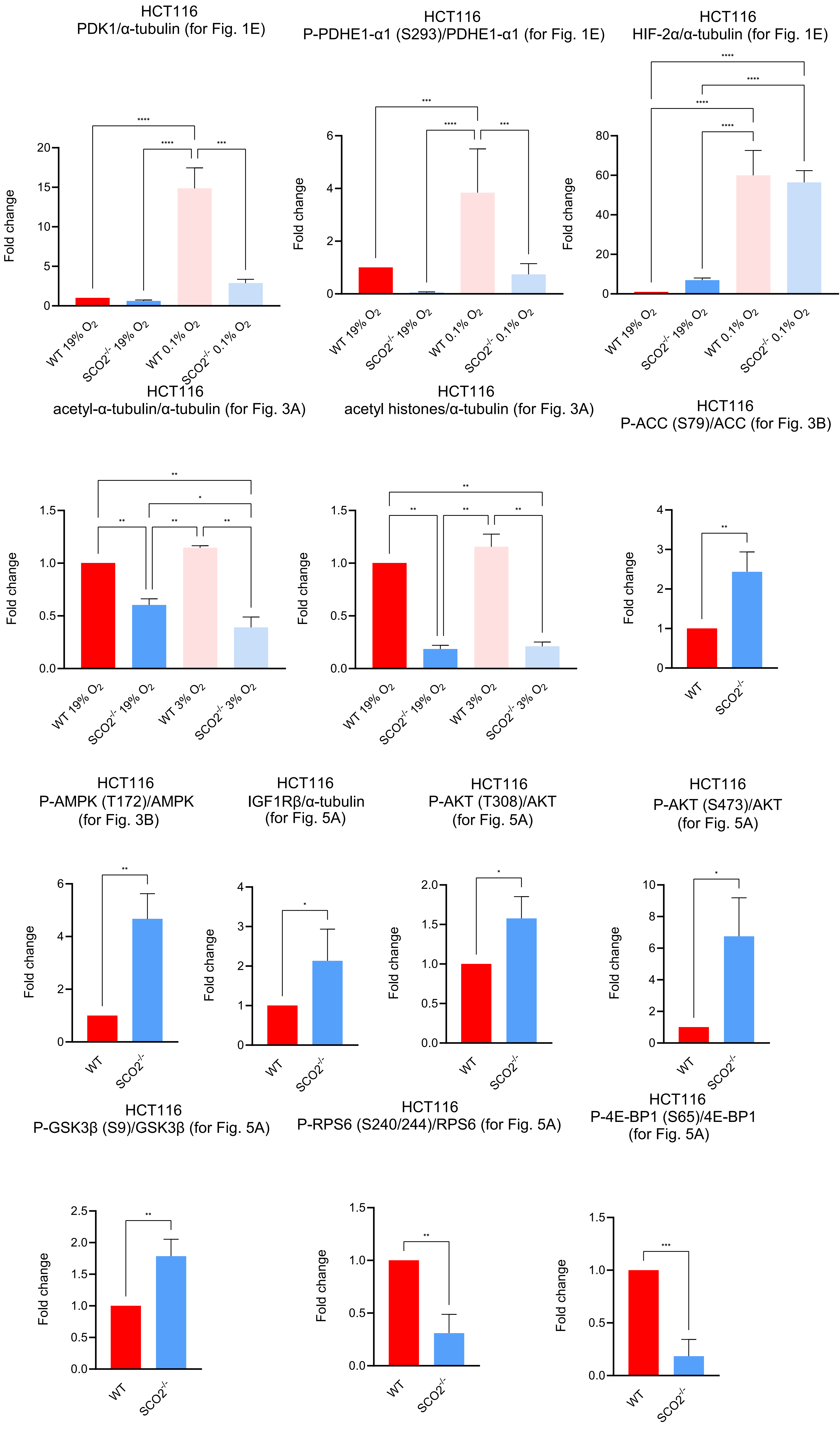

Supplemental Figure 9

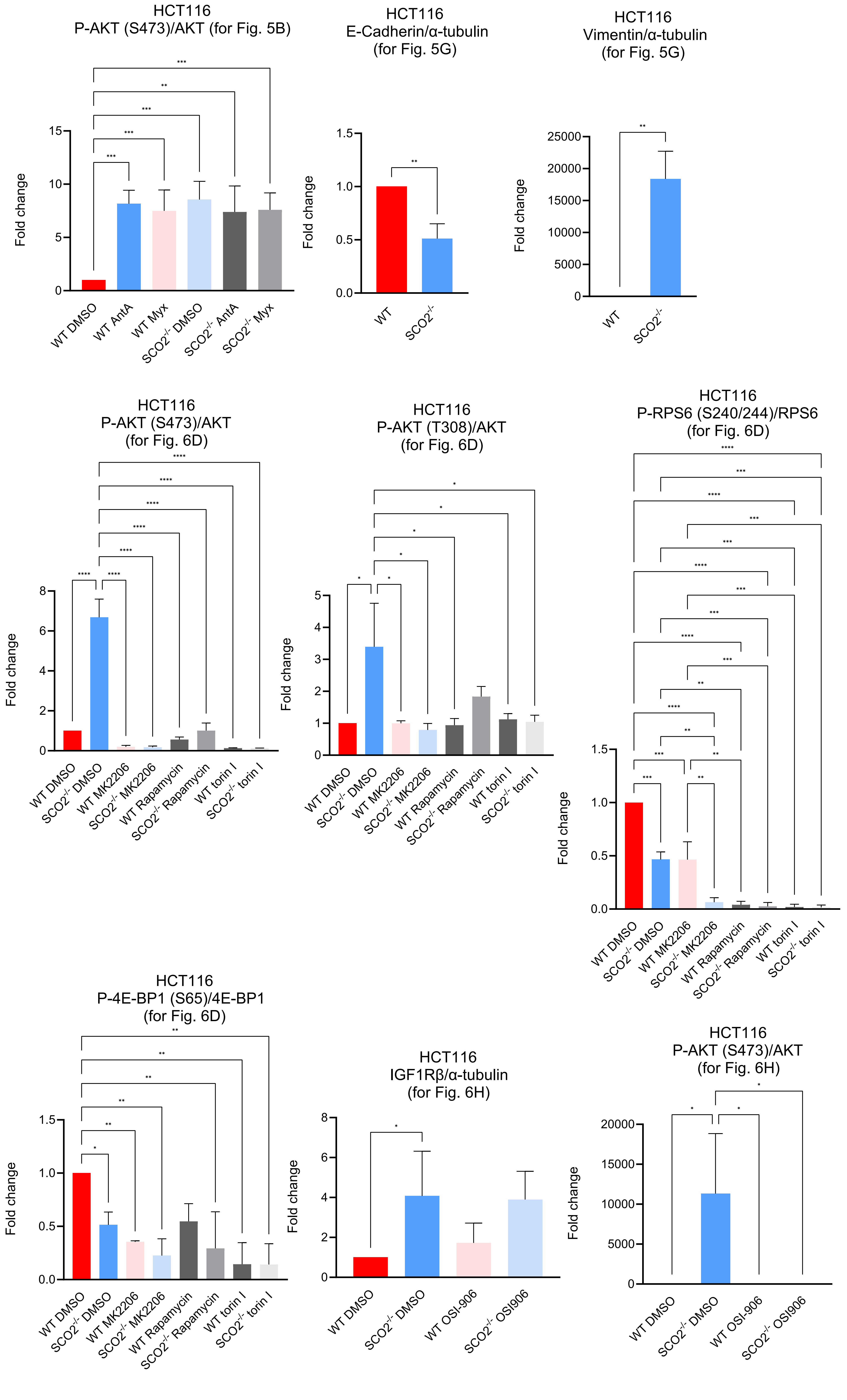

Supplemental Figure 9

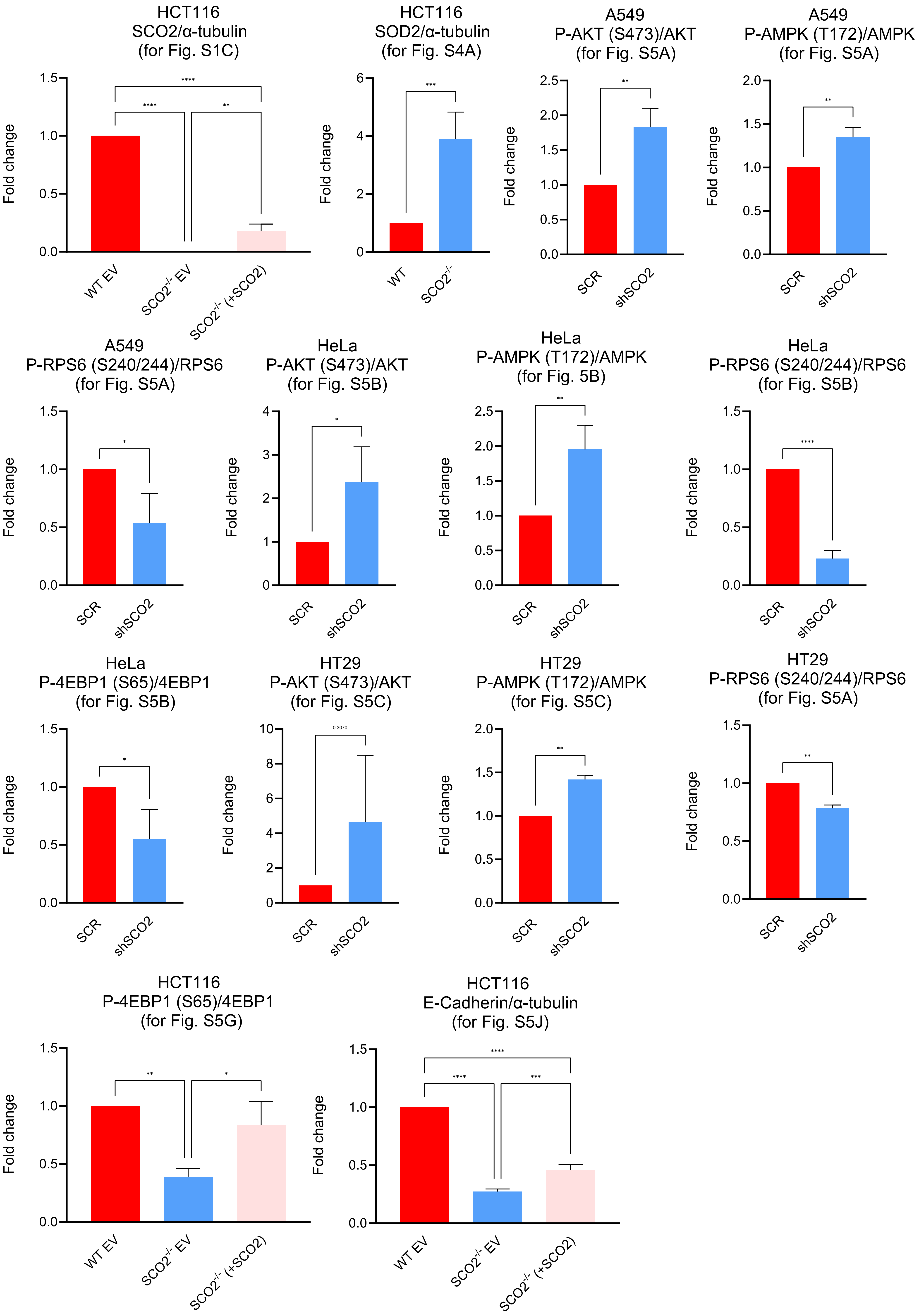

Supplemental Figure 9

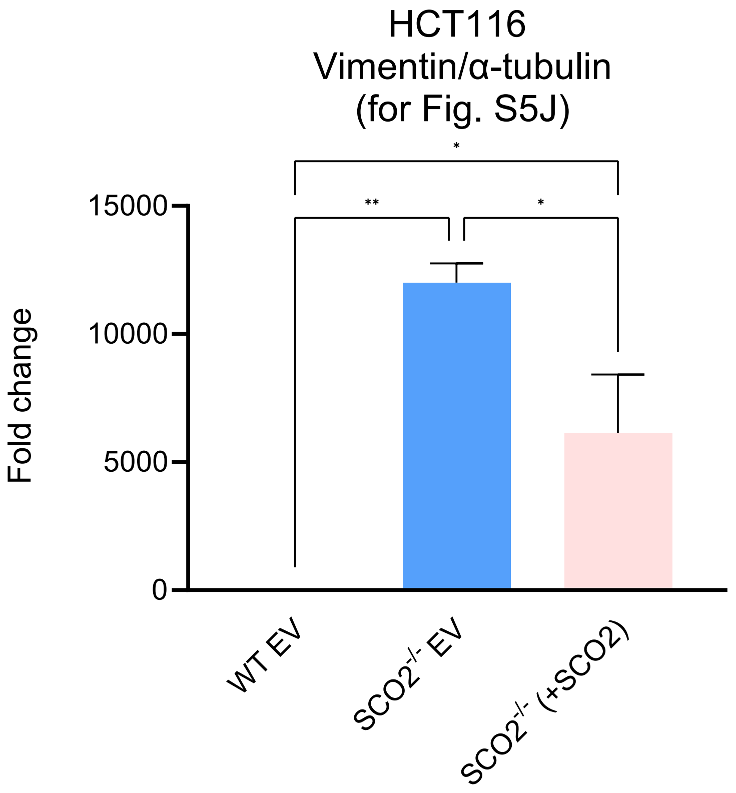

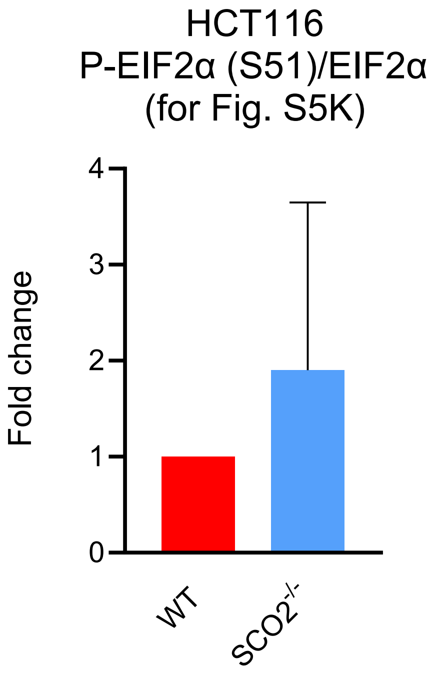

Supplemental Figure 9

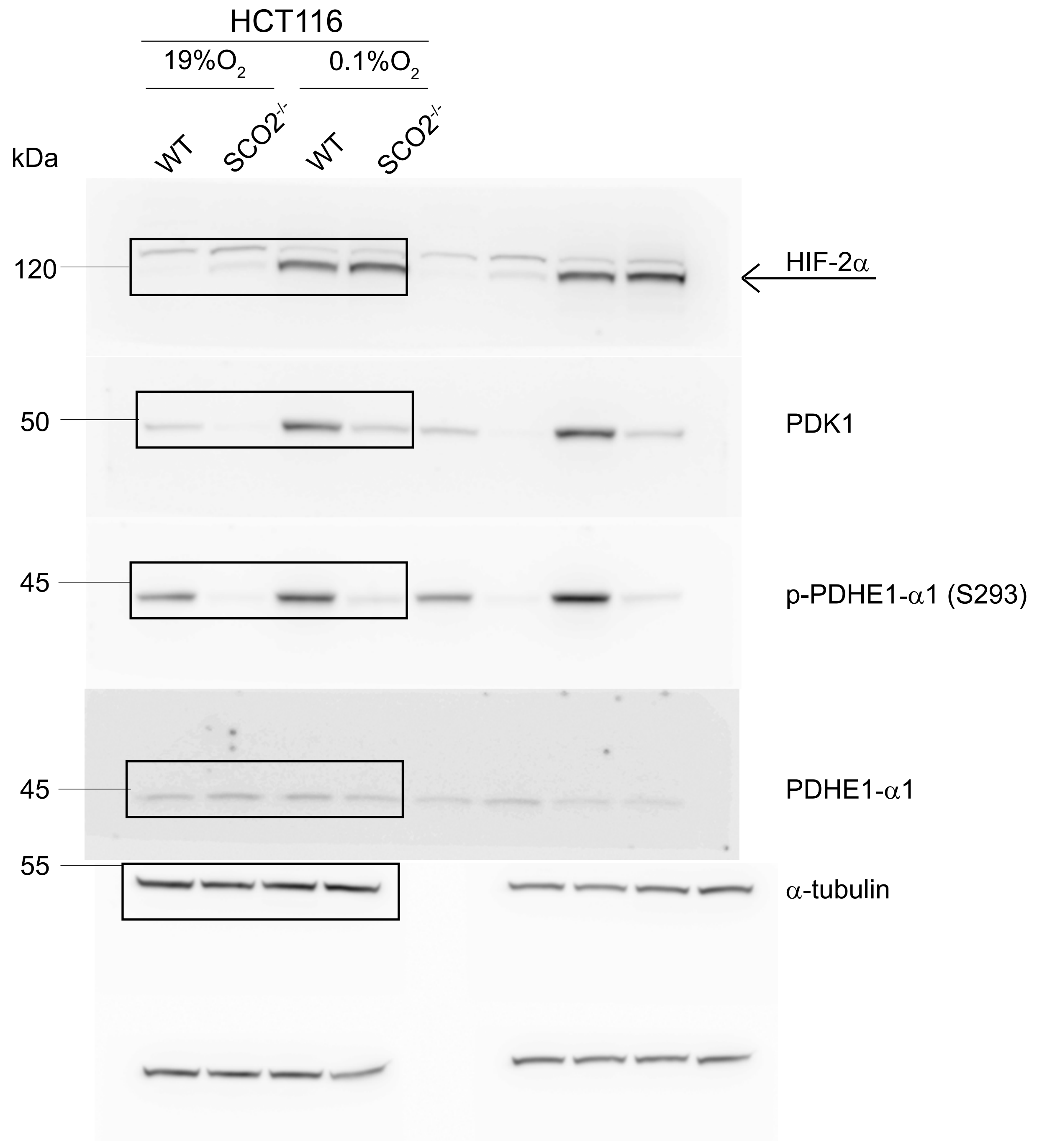

(for Fig. 1E)

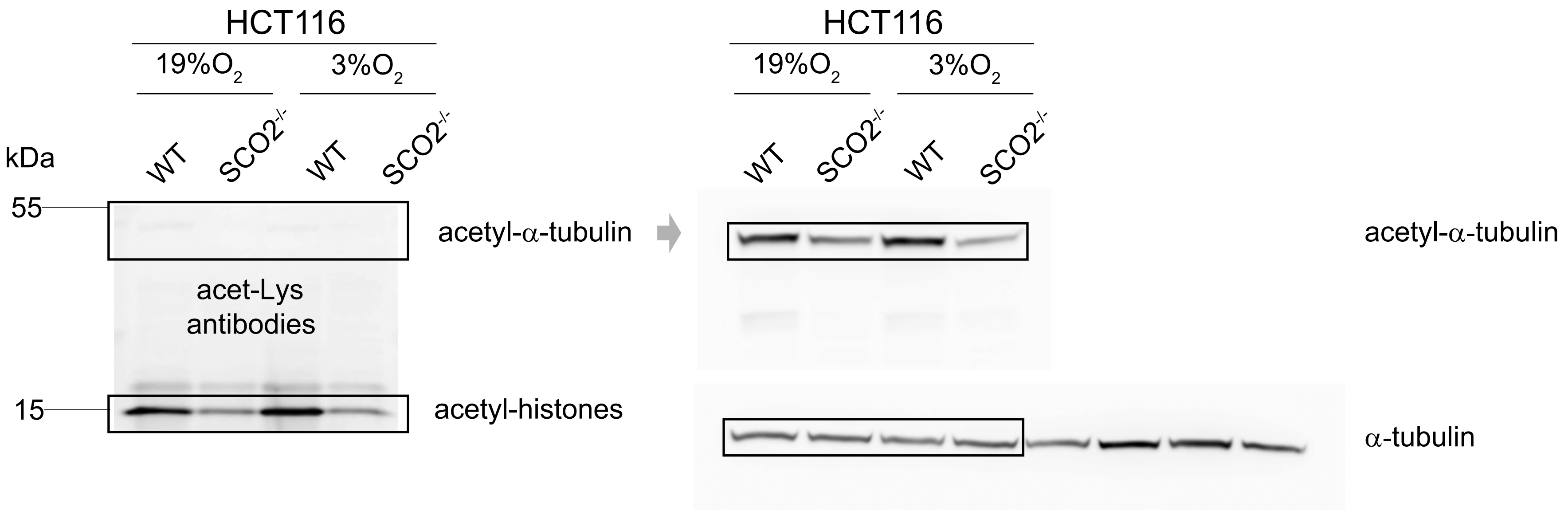

(for Fig. 3A)

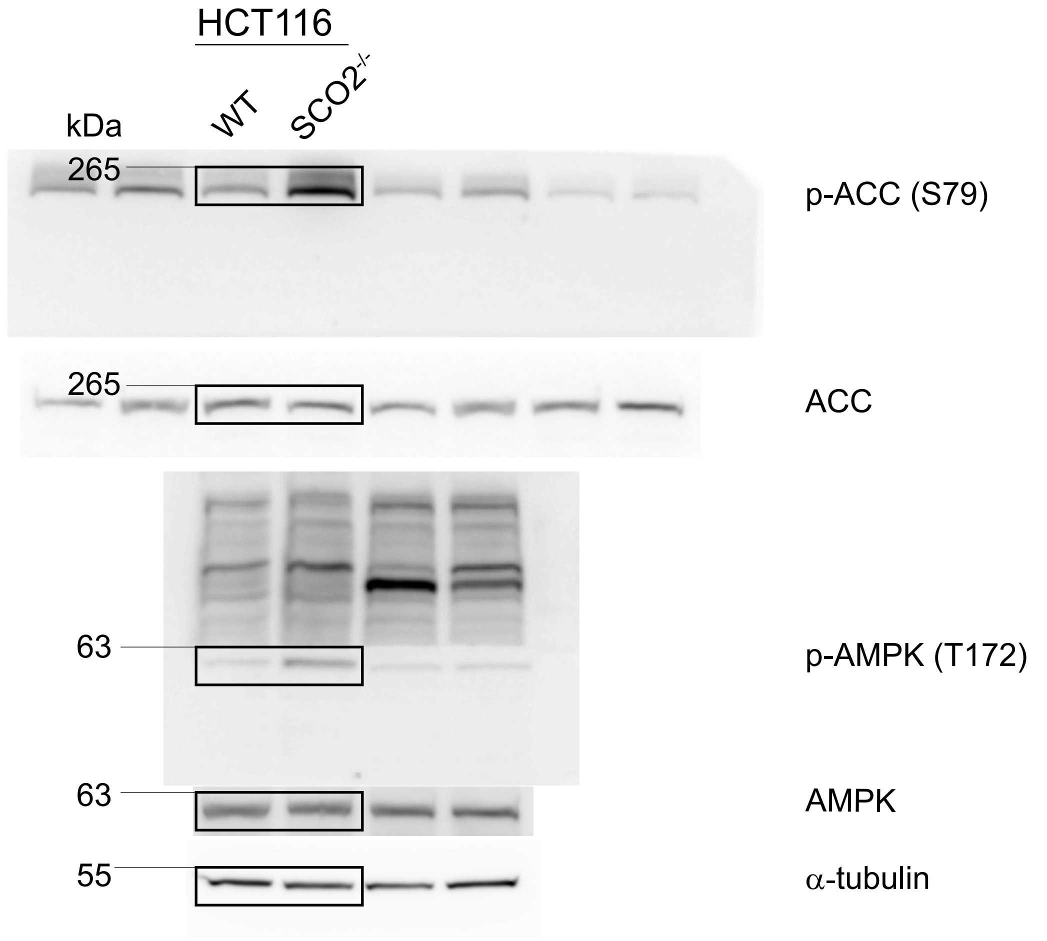

(for Fig. 3B)

Supplemental Figure 10

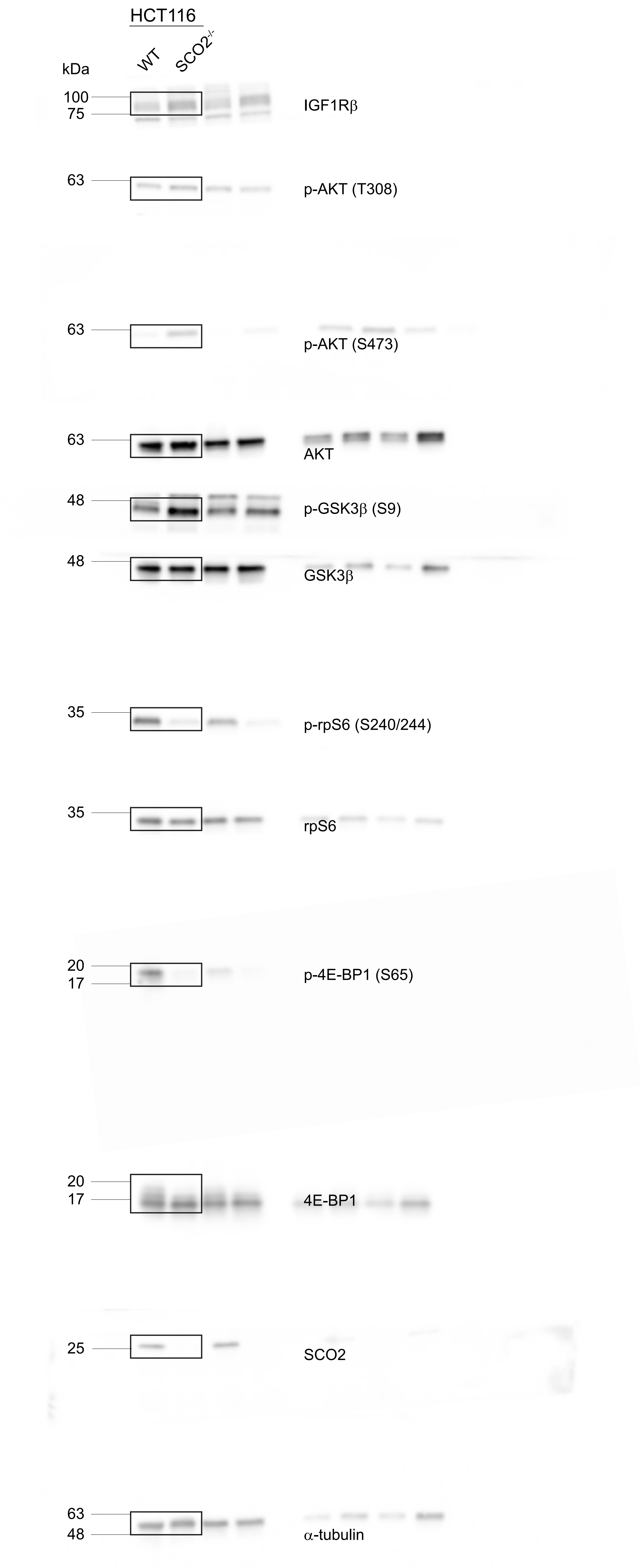

(for Fig. 5A)

Supplemental Figure 10

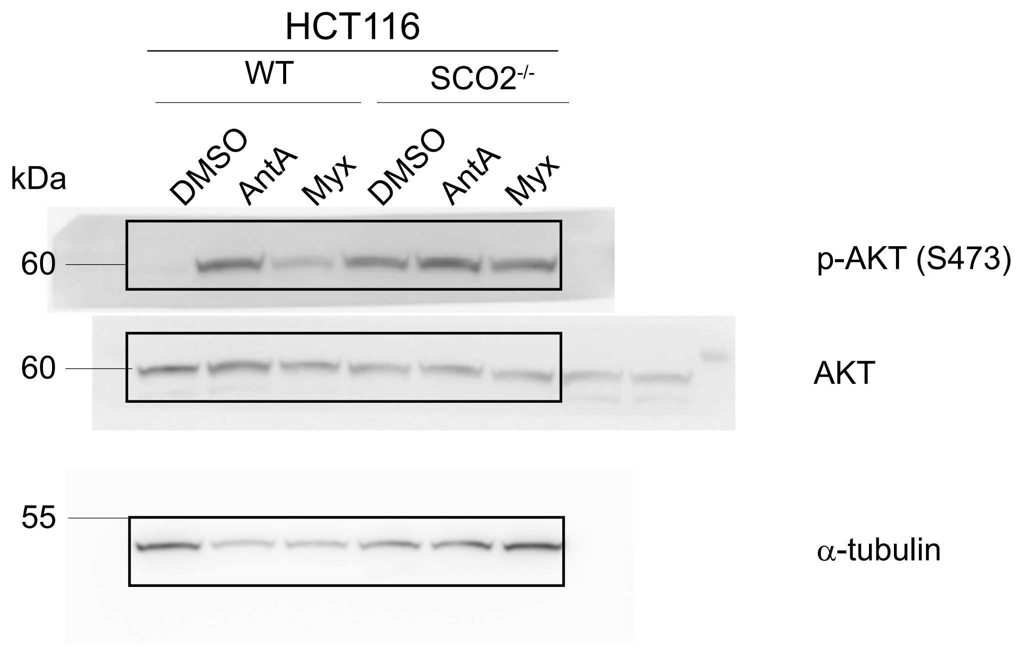

(for Fig. 5B)

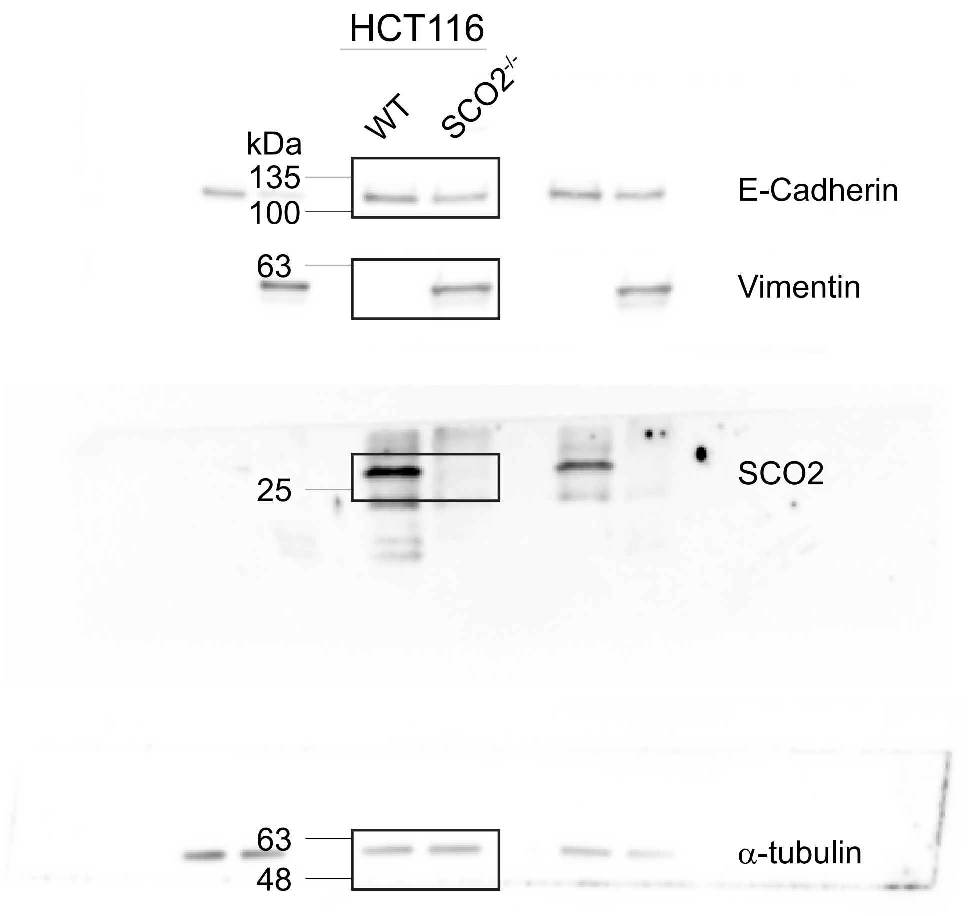

(for Fig. 5G)

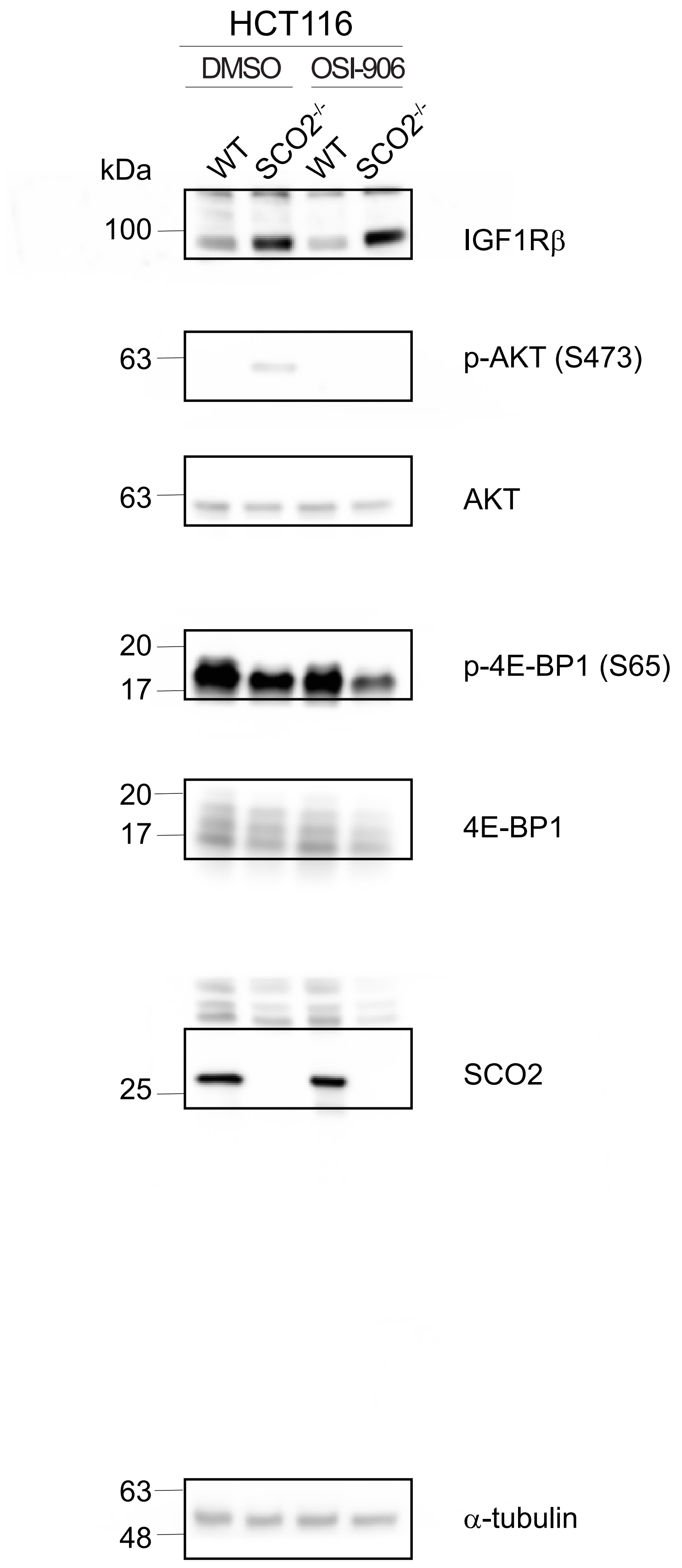

(for Fig. 6H)

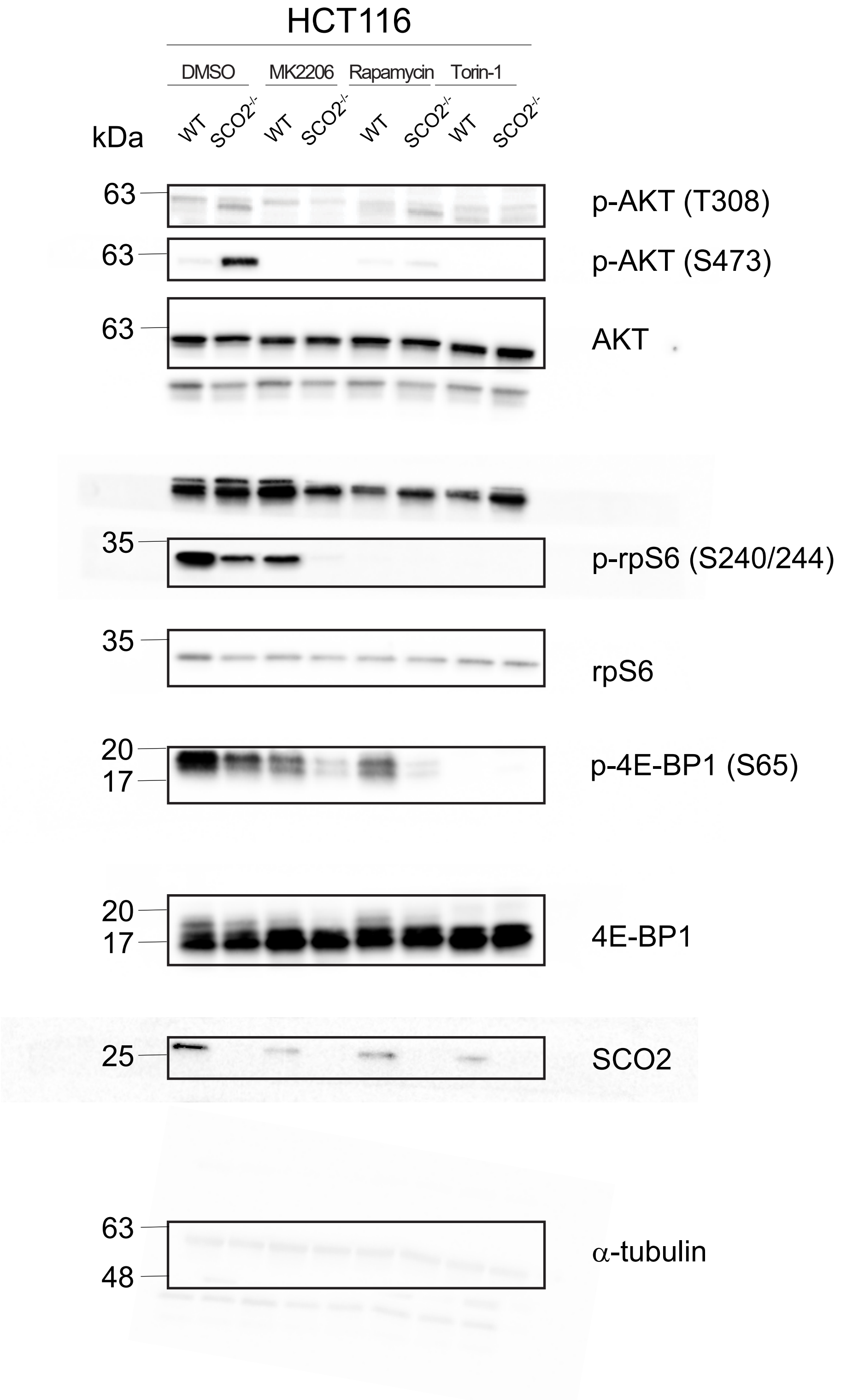

(for Fig. 6D)

Supplemental Figure 10

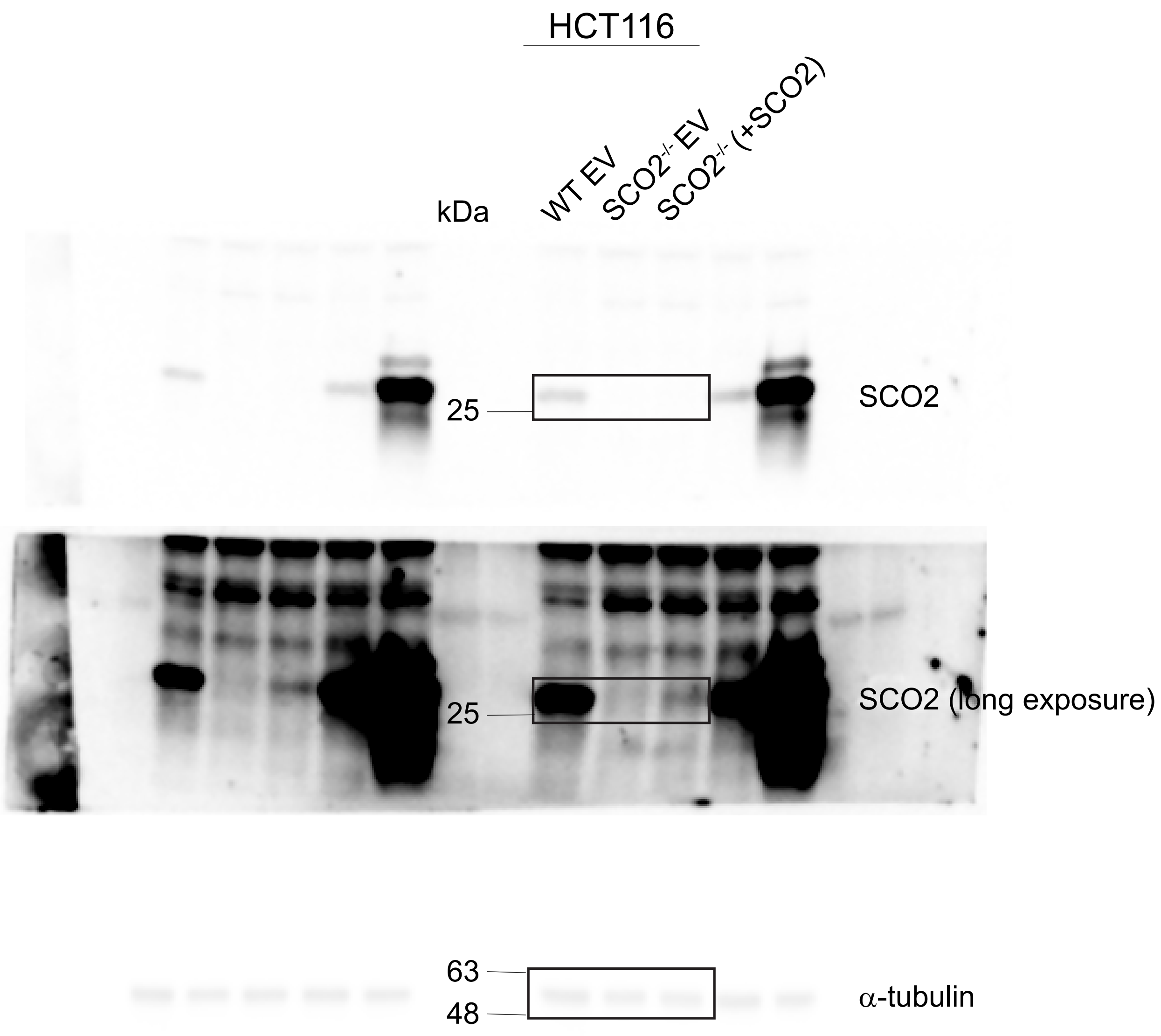

(for Fig. S1E)

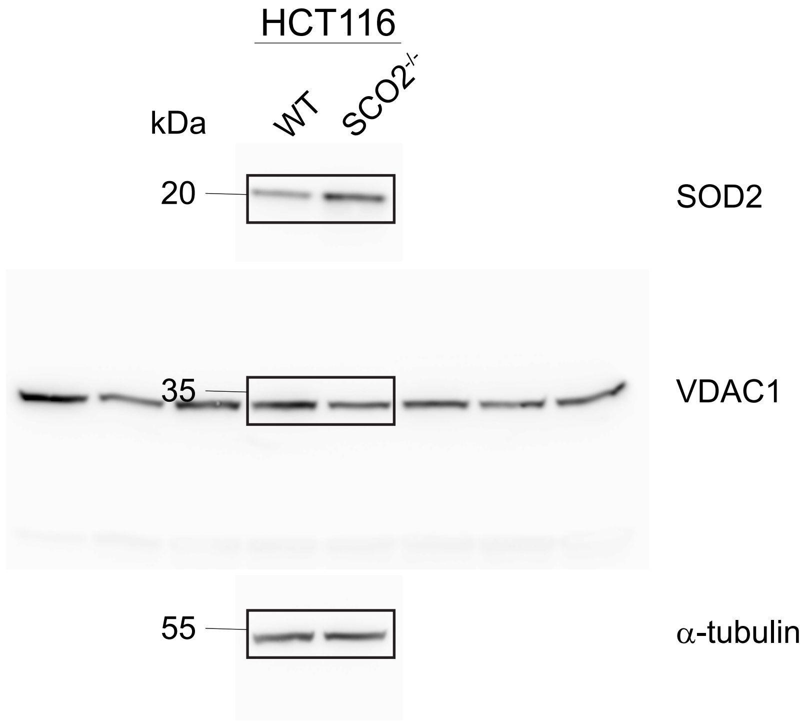

(for Fig. S4A)

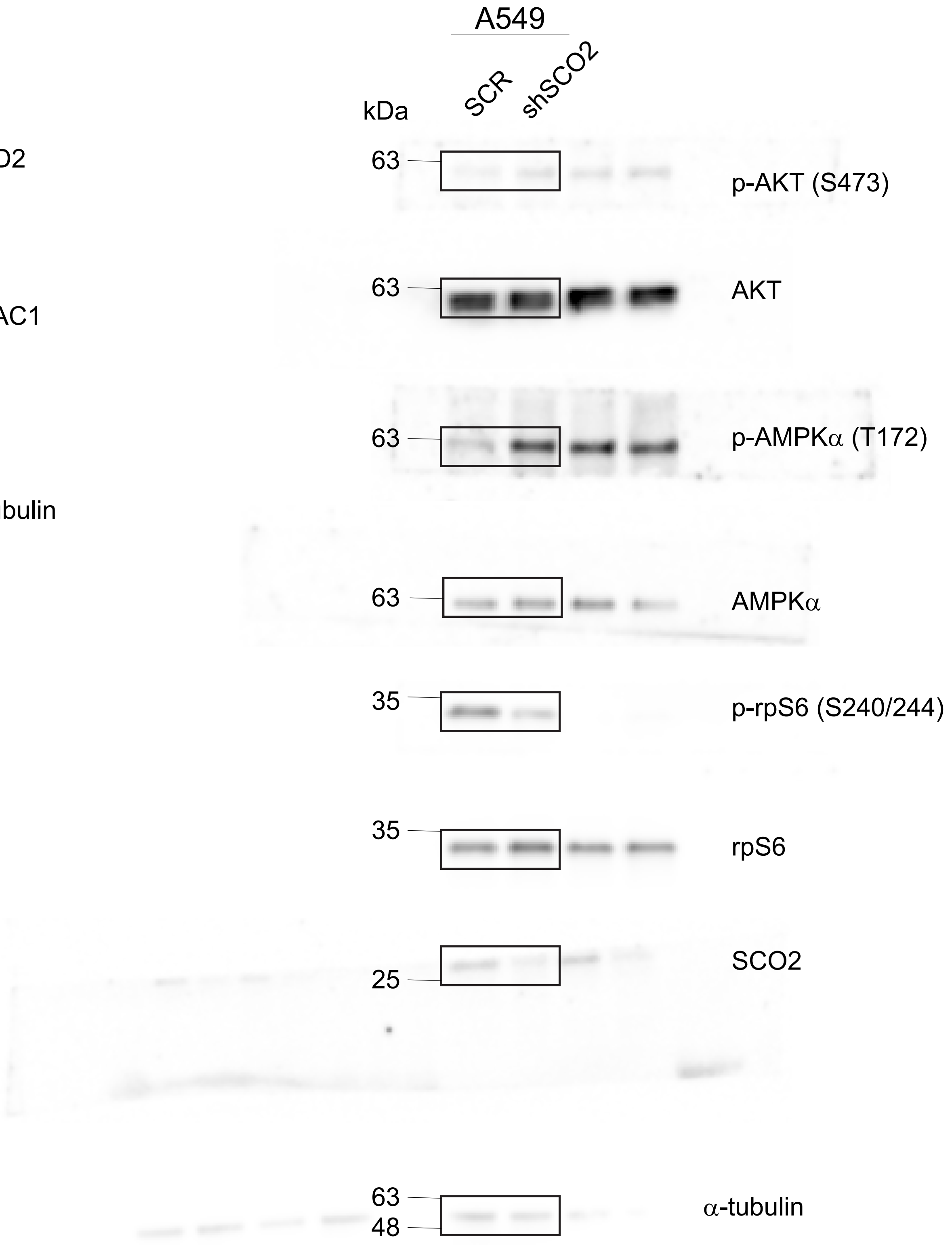

(for Fig. S5A)

Supplemental Figure 10

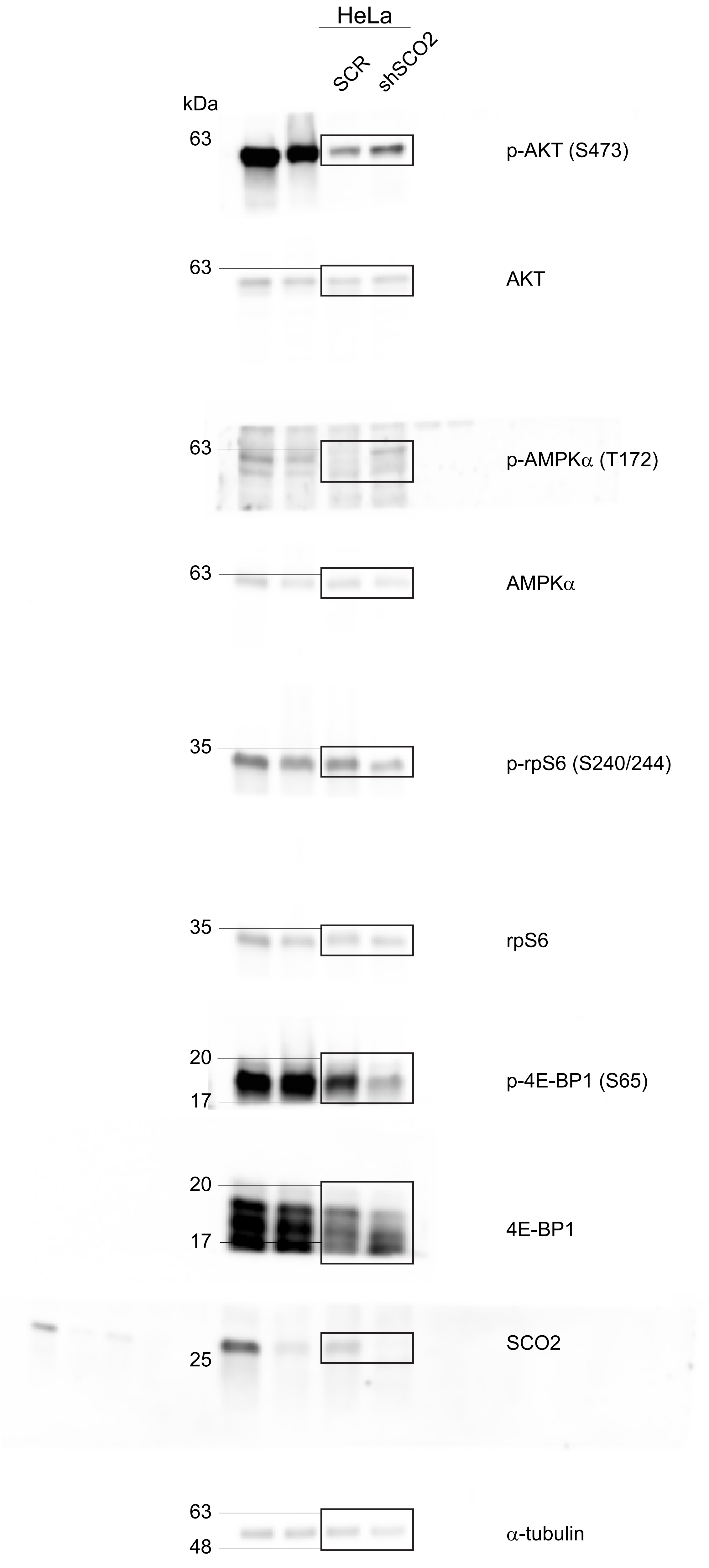

(for Fig. S5B)

Supplemental Figure 10

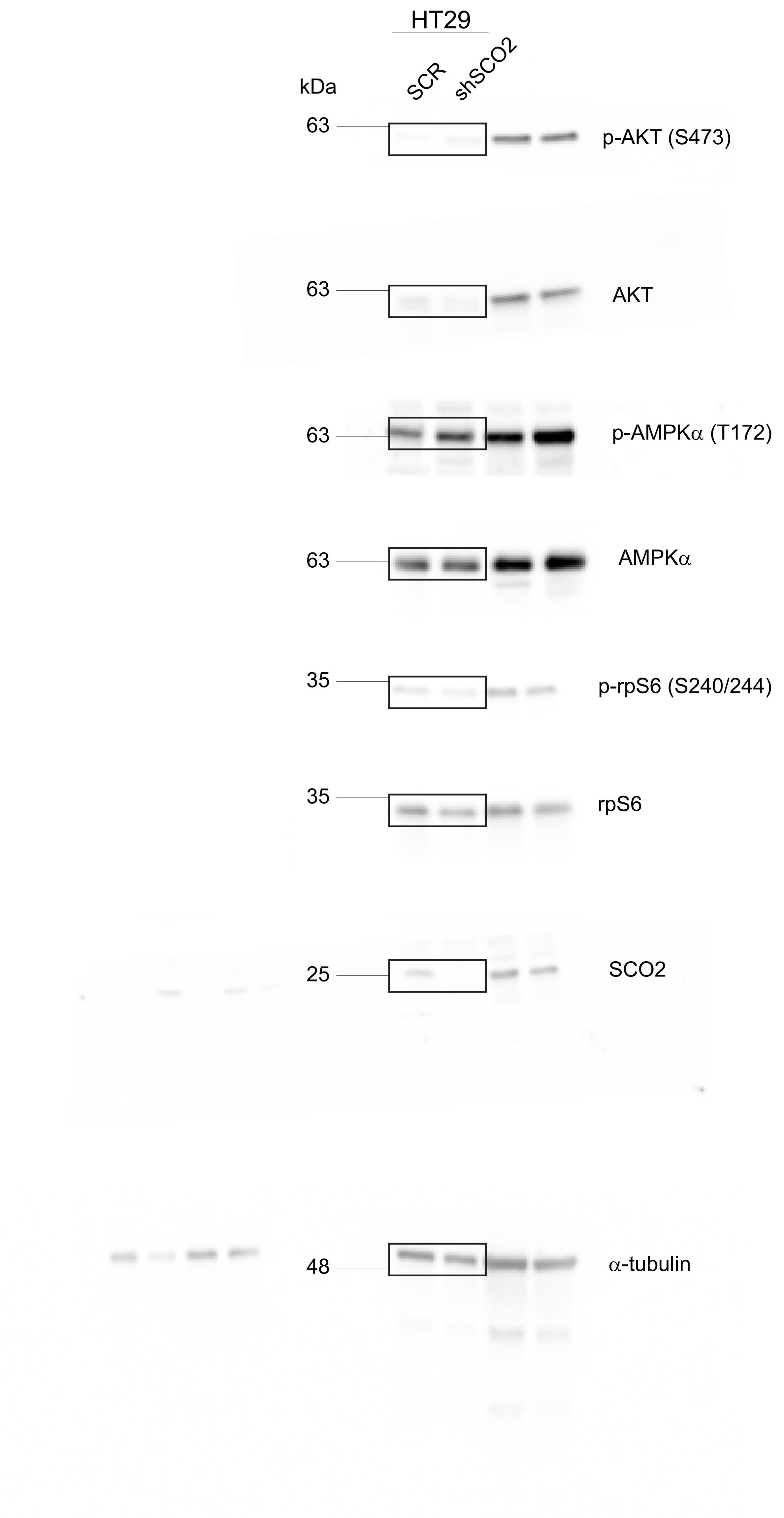

(for Fig. S5C)

Supplemental Figure 10

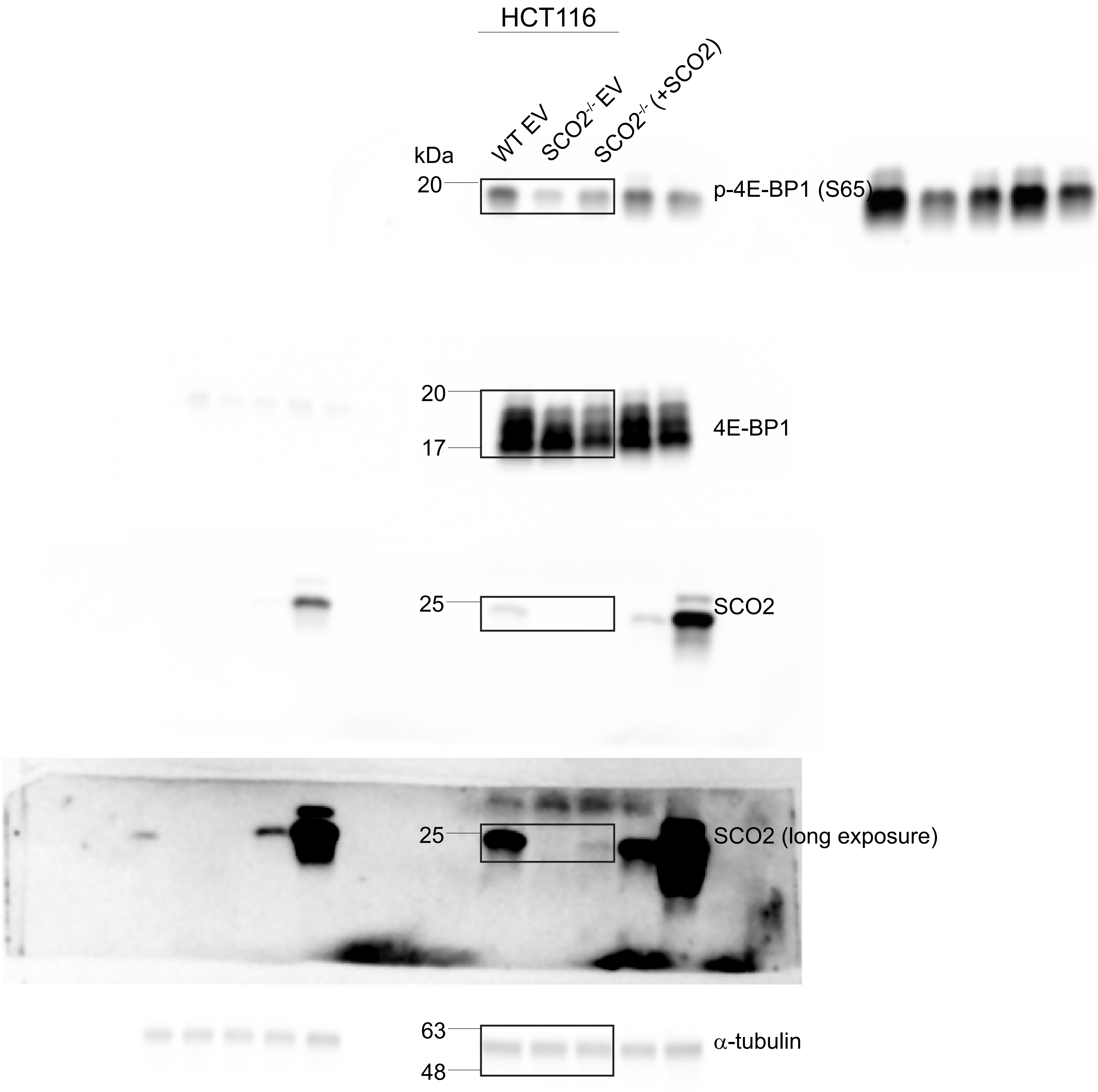

(for Fig. S5G)

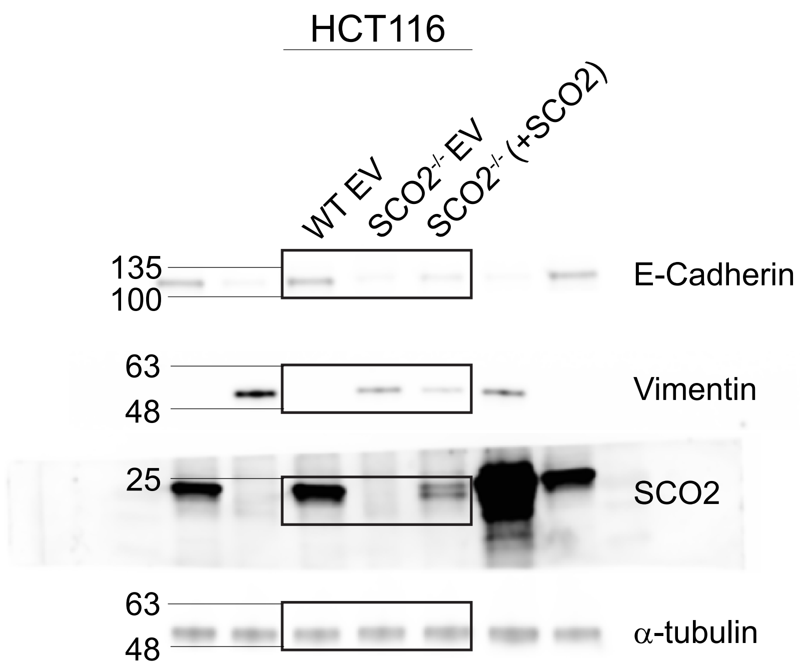

(for Fig. S5J)

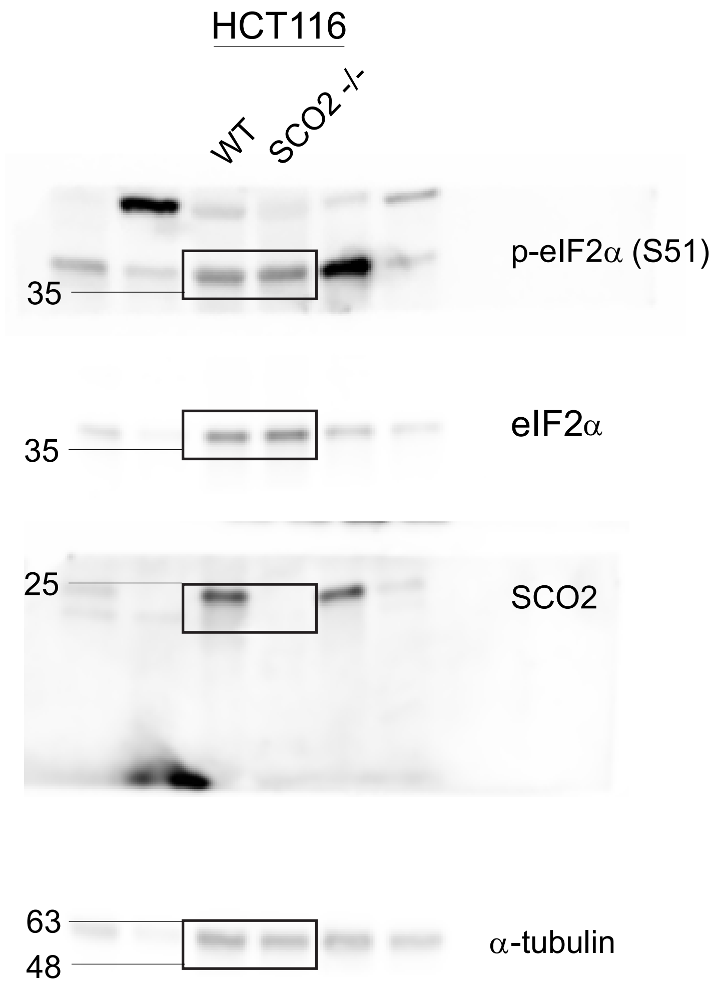

(for Fig. S5K)

Supplemental Figure 10
